# Use of Ionic Liquids in Protein and DNA Chemistry

**DOI:** 10.3389/fchem.2020.598662

**Published:** 2020-12-23

**Authors:** Shashi Kant Shukla, Jyri-Pekka Mikkola

**Affiliations:** ^1^Technical Chemistry, Department of Chemistry, Chemical-Biological Centre, Umeå University, Umeå, Sweden; ^2^Industrial Chemistry and Reaction Engineering, Department of Chemical Engineering, Johan Gadolin Process Chemistry Centre, Åbo Akademi University, Åbo-Turku, Finland

**Keywords:** ionic liquid (IL), DNA, protein, Hofmeister series, intermolecular interaction, circular dichroism, double-helical structure, salting phenomenon

## Abstract

Ionic liquids (ILs) have been receiving much attention as solvents in various areas of biochemistry because of their various beneficial properties over the volatile solvents and ILs availability in myriad variants (perhaps as many as 10^8^) owing to the possibility of paring one cation with several anions and *vice-versa* as well as formulations as zwitterions. Their potential as solvents lies in their tendency to offer both directional and non-directional forces toward a solute molecule. Because of these forces, ionic liquids easily undergo intermolecular interactions with a range of polar/non-polar solutes, including biomolecules such as proteins and DNA. The interaction of genomic species in aqueous/non-aqueous states assists in unraveling their structure and functioning, which have implications in various biomedical applications. The charge density of ionic liquids renders them hydrophilic and hydrophobic, which retain intact over long-range of temperatures. Their ability in stabilizing or destabilizing the 3D-structure of a protein or the double-helical structure of DNA has been assessed superior to the water and volatile organic solvents. The aptitude of an ion in influencing the structure and stability of a native protein depends on their ranking in the Hofmeister series. However, at several instances, a reverse Hofmeister ordering of ions and specific ion-solute interaction has been observed. The capability of an ionic liquid in terms of the tendency to promote the coiling/uncoiling of DNA structure is noted to rely on the basicity, electrostatic interaction, and hydrophobicity of the ionic liquid in question. Any change in the DNA's double-helical structure reflects a change in its melting temperature (*T*_m_), compared to a standard buffer solution. These changes in DNA structure have implications in biosensor design and targeted drug-delivery in biomedical applications. In the current review, we have attempted to highlight various aspects of ionic liquids that influence the structure and properties of proteins and DNA. In short, the review will address the issues related to the origin and strength of intermolecular interactions, the effect of structural components, their nature, and the influence of temperature, pH, and additives on them.

## Introduction

The energetics of a biological reaction/process change upon perturbing the solvent systems around it (Yancey et al., 1982). Molecular solvents, depending upon its nature, might offer various interactions ranging from dipole-dipole, electrostatic, van der Waals, hydrogen bonding, hydrophobic interactions, and so on. However, it is impossible to accommodate all the said interactions in a single molecular solvent. Water, despite its recognition as a universal solvent, offers only dipole-dipole, hydrogen bonding, and hydrophobic interactions for a solute. Almost all basic biological entities necessarily require a medium for their stabilization and functioning. Out of various basic biological candidates, we are, in particular, addressing the proteins and DNA in detail. As both proteins and DNA possess basic moieties, electrostatic, and hydrophobic centers, they require a medium that has all these interactions for their stabilization, functioning, long-term storage, and separation.

**Figure 1:**
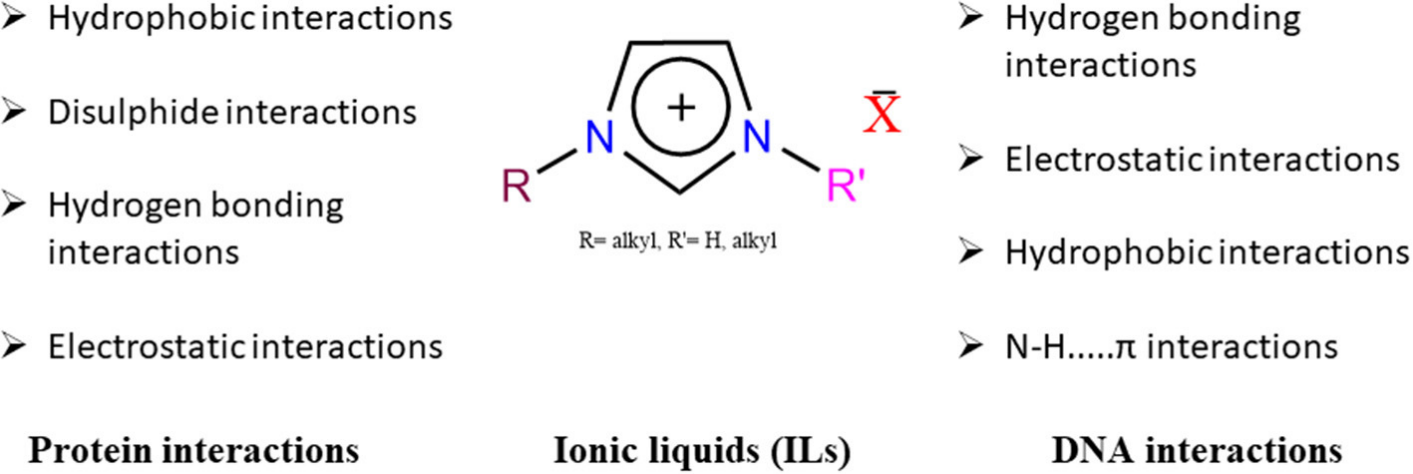
Interactions of a typical ionic liquids with proteins and DNA. Images in the backgroud are taken from the google.

In quest of the “green” solvents, the past few decades have witnessed the upsurge of ionic liquids (ILs) as a suitable replacement of volatile organic solvents (VOCs) in various applications. Ionic liquids (IL) are typically liquid at room temperature and mainly consists of asymmetric organic cation and organic/ inorganic anion (Wilkes and Zaworotko, 1992). Ionic liquids are classified as protic ionic liquids (PILs) and aprotic ionic liquids (AILs), depending on the quaternization of base by proton (H^+^) or alkyl group (-R), respectively. These two ionic liquids differ a lot in terms of the activity and strength of the directional and non-directional forces between ions. Protic ionic liquids possess higher strength of Coulomb- and hydrogen bonding-interactions as compared to their aprotic counterparts and resembles closely to water, owing to their extensive network of hydrogen bonding (Fumino et al., 2009) Similar to PILs are Brønsted acidic ionic liquids, in which protons occupy either the cation or the anion (not necessarily on the cation as in protic ionic liquids), and therefore, they are categorized with the protic/aprotic ionic liquids. The reputation of ionic liquids in several fields is because of their attractive properties such as significantly low vapor pressures, high thermal stability, wide liquidus range, non-flammability, and recyclability (Welton, 1999; Weingärtner, 2008; Castner and Wishart, 2010; Hayes et al., 2015; Egorova et al., 2017). Their relevance in various chemical and biological processes is owing to their ability to interact through the variety of forces toward a solute. The physicochemical properties of ionic liquids depend on the coordination of asymmetric cation with a symmetric or asymmetric organic/inorganic anion (Rogers and Seddon, 2003). Depending on the charge density of anion, ionic liquids behaves as hydrophilic or hydrophobic in water and therefore can react with both polar and non-polar solutes. Besides, a long alkyl chain either on cation or on anion also imparts hydrophobic character to the ionic liquid. Ionic liquids in neat and in aqueous state affects the structure and stability of proteins and hence are being tried as a potential media in storage and crystallization. Despite the huge interests in ionic liquids, the molecular mechanism involved in protein-ionic liquid are not yet fully comprehended. The availability of different kinds of ionic liquids and various features of proteins make the generalization about the intermolecular interactions very difficult. Addition of water though alleviates the solubility problem and maintains the structure over a longer time period; however, it complicates the understanding of the intermolecular interactions between the ionic liquid and biomolecules as water also undergoes strong hydrogen bonding with both species. The physical parameters for the aqueous ionic liquids, however, cannot be accounted for by the screened forces of the ionic liquid toward the biomolecule. In this regard, different solvation models have been employed to derive the interaction parameters that explain the stabilizing/destabilizing behavior of aqueous ionic liquid systems toward biomolecules. The structure and stability of the DNA's double-helical structure also get affected in the dilute solution of an ionic liquid. Figure 1 and Table 1 shows the interactions between ionic liquids and protein and DNA molecules.

**Table 1:** Summary of interactions between ionic liquids and biomolecules and responsible functionality/moiety.

**Type of interactions**	**Responsible group/moiety on ionic liquids**
Hydrophobic interactions	Hydrophobic surfaces of protein/DNA and alkyl chain on cation
Disulphide (S-S) interactions	Anion and protein linkage
Hydrogen bonding interactions	Anion and electron donor moiety on protein and DNA bases
Electrostatic interactions	Cation with P=O bonds on DNA strands and anion with negatively charged surface on protein
NH—π interactions	Aromatic cation and DNA bases

There are some excellent review articles available on the use of ionic liquids in the stabilization and functioning of protein and DNA. The review article by Schindl et al. thoroughly discusses the use of different classes of ionic liquids in the dissolution and processing of different types of proteins (Schindl et al., 2019). The review by Gough et al. presents a critical discussion on the use of ionic liquids as potential media for protein and polysaccharide biopolymers fibrillation by different fiber fabrication methods (Gough et al., 2020). The topical review by Smiatek presents detailed discussion about the role of ionic liquids as solvents in deciding the fate of protein to the native and denatured state by using a preferential binding model, where the strength of ions in influencing the protein dynamics is given in terms of the value of preferential binding coefficient (Smiatek, 2017). The role of water on the self-aggregation of ionic liquids is also discussed with recent experimental and theoretical findings in light of the binding coefficient values. Wakayama et al. presented a review based on various model parameters to construct the enzyme-derived catalyst in the production of important chemicals (Wakayama et al., 2019). The review by Saha and Mukherjee enlists the simulation results on the effect of water in altering the structural and dynamical properties of ionic liquids and biomolecules present in it (Saha and Mukherjee, 2018). Reslan and Kayser compiled all the experimental and theoretical results available on the ionic liquid-protein interactions and discussed the effect of toxicity and viscosity on it (Reslan and Kayser, 2018). Oprzeska-Zingrebe and Smiatek discussed the relative importance of the aqueous ionic liquids and standard co-solutes in stabilizing or destabilizing the protein and DNA structure (Oprzeska-Zingrebe and Smiatek, 2018). The extent of stabilization/destabilization by the ionic liquids are discussed in terms of the enthalpic and entropic contributions obtained from the Gibbs free energy change. The review by Schröder gives a detailed account of the various properties of ionic liquids that could affect the protein stability and crystallization in view of the various classical theories (Schröder, 2017). Contrary to the multiple reviews available on the behavior and functioning of protein in ionic liquid systems, information on DNA stabilization in ionic liquid systems is scarce. The review by Zhao though gives a good account of the use of ionic liquids on the DNA stabilization in terms of various specific and non-specific-interactions between them (Zhao, 2015).

In the current review, we aim to discuss the role of intermolecular interactions of ionic liquids on the structure and functioning of various proteins and DNA. These interactions are discussed in terms of the change of cation, anion, length of alkyl chain, and presence of functional group on them. A brief introduction of various theories/concepts in the protein stabilization by electrolytes solutions and their utility in case of ionic liquids is presented. Besides the variation of structural components, the influence of the reaction conditions such as pH, temperature, and effect of water on the intermolecular interactions are also described. The importance of ionic liquid-biomolecules interactions on, stability, solubility and separations are also covered. The negative impact of toxicity of ionic liquids on the storage and functioning of biomolecules and recyclability is also taken into the consideration.

## Ion-Protein Interactions

We begin this section with a brief discussion about the solvent property such as polarity and hydrophobicity that have implications on the protein and DNA chemistry. Subsequently, various theories/concepts related to the ion-protein interactions like the Hofmeister series, Collins law of matching water affinity, Jones-Dole coefficient, and the influence of ionic strength will be discussed. The validity and deviation of Hofmeister series is also extended to the ionic liquids. At the end, specific role of water on the protein-ionic liquid interactions is discussed.

Nearly all proteins contain a lower or higher amount of sulfur (3–6%) in their structures that impart rigidity to the 3-D structure owing to the formation of disulfide (-S-S-) bonds (Simpson and Crawshaw, 2002). Besides, various inter- and intra-molecular hydrogen bonds in protein also contribute to the rigidity and hence posing a challenge in their processing. Therefore, a solvent/mixture whose components outshine the –S-S- and inter- and intra-molecular interactions are desired for protein processing. The use of molecular solvents in protein processing has lingered with various issues. They poses a risk of volatility, corrosiveness, environmental impact, recycling, and renewability (Idris et al., 2013). In addition to this, their inapplicability in multi-step processes and the degradation of proteins are the prime issues related to solvent mixtures. Due to these shortcomings, there has been a growing demand to develop a new solvent that can fulfill the entire solvent properties for protein processing. The solvation behavior among different classes of ionic liquids is not similar and can be assessed from the polarity parameters and hydrophobicity. We would discuss in detail the characteristics of ionic liquids such as polarity, hydrophobicity and potential of ion, that come-in-aid to stabilize the protein structure.

### Polarity

The solvation capability of a solvent is best to describe in terms of a single polarity parameter that accounts for the entire specific- and non-specific-interactions (Reichardt, 2005). There are various scales of solvent polarity such as static dielectric constant (ε_*r*_), Reichardt polarity scale (*E*_*T*_(30)), refractive index (η), and permanent dipole moment (μ) (Reichardt, 1994). Out of these, the static dielectric constant and Reichardt polarity scales are largely utilized to account for the “solvation effects/polarity.” The non-specific electrostatic interactions are measured by dielectric spectroscopy (Huang and Weingärtner, 2008). The protic ionic liquids possesses a higher dielectric constant whereas the polarity of aprotic ionic liquids is in the range of lower alcohols. In the case of ionic liquids, which possess charged ions, static conductivity σ (0) dominates in the dielectric constant. However, it is noted that the dielectric constant of ionic liquids is lower than that measured by the diffusion coefficient due to the interaction of ions itself rather than the neutral ion-pair. The mobility of ions poses a problem in the measurements of dielectric constant (Krüger et al., 2010).

The “Reichardt polarity scale” is devoid of any complications and determines all the specific, non-specific, π-π and dipole-dipole interactions of ionic liquids and molecular solvents (Reichardt, 2008). In this method, any change in the electronic transition due to the transfer of the Reichardt's dye from non-polar to polar medium is measured by UV-visible spectrophotometer and is represented as the electronic transition energy (*E*_*T*_(30)) measured in kcal.mol^−1^. Kamlet, Abboud, and Taft proposed the idea of a complementary polarity scale using a set of dyes that are known as polarity index (π^*^), hydrogen bond donor acidity (α), and hydrogen bond acceptor basicity (β) (Kamlet and Taft, 1976; Taft and Kamlet, 1976; Kamlet et al., 1977). α denotes the hydrogen bond donor capacity and is a characteristic of cation whereas the β accounts for the hydrogen bond acceptor basicity and depends on the anion. The strength of electrostatic interaction between ions of ionic liquid is determined in terms of the polarity index, π^*^. Protic ionic liquids possess higher α and lower β than their aprotic counterparts. The interaction of cations and anions of ionic liquids to the protein and DNA depends on the charge and acidity/basicity of the interacting sites on biomolecules.

### Hydrophobicity

Hydrophobicity is a phenomenon in which a non-polar solute molecule tends to stay away from water (Zhao, 2006). It has a crucial role in protein solubilization and enzyme functioning. The low charge density on protein and enzymes makes them hydrophobic in water. A saturated 1-octanol-water mixture is used to measure the hydrophobicity of a solute in terms of the partition coefficient shown as log P, which denotes the partitioning of solute in 1-octanol as compared to water. Solvents with high log *P* are more hydrophobic compared to those with lower log *P*-values. Ionic liquids possess lower log *P*-values than short-chain alcohols. For example, 1-butyl-3-methylimidazolium with acetate, nitrate, and hexafluorophosphate anions have log *P*−2.8,−2.9, and−2.4, respectively than ethanol−0.24. Thus, ionic liquids are less hydrophobic or more hydrophilic than ethanol. The tethering of longer alkyl chain on cation or anion increases the log *P* (Yamamoto et al., 2011). Though hydrophobicity was found significant in enzyme catalysis, it inversely affects the solubility of the protein (Laane et al., 1987). In the case of proteins, hydrophobicity overshadows the basicity of anion or coulombic interactions (Kaar et al., 2003).

### Effect of Ion on Protein Solubility: Hofmeister Series

Ion-induced effects are universal in chemistry and biology and emerged through the alteration in the hydrogen bonding network of water (Hofmeister, 1888). These effects were important in protein and enzyme stabilization and crystallization but the fundamental mechanism behind these effects were not well-understood (Collins, 2004; Kunz et al., 2004; Broering and Bommarius, 2005; Nostro et al., 2005). In 1888, Franz Hofmeister arranged several cations and anions into their capability of precipitating the egg white protein (Hofmeister, 1888). For a given cation, anions' coagulating power was in the order,

$$\begin{array}{l} {\text{SO}}_{4}^{2-}>\;{\text{HPO}}_{4}^{2-}>\;{\text{F}}^{-}>{\text{CH}}_{3}{\text{COO}}^{-}>\;{\text{Cl}}^{-}>\;{\text{Br}}^{-}>{\text{NO}}_{3}^{-}\\ >\;{\text{I}}^{-}>{\text{ClO}}_{4}^{-}>\;{\text{SCN}}^{-} \end{array}$$

A reverse of the above ordering was noted operative in predicting the precipitating power of anions though not universally obeyed. Afterwards several experimental and theoretical reports claimed that protein stabilization in an electrolytic medium depends on the specific solute-ion interactions (Omta et al., 2003a,b; Zhang et al., 2005). Later, Gurney, Washbaugh and Collins, and Green explained the confusion over the validity of the Hofmeister series (Gurney, 1953; Collins, 2004, 2006).

Gurney classified salts as structure–maker (kosmotropic) and structure-breaker (chaotropic) depending on their ability to strongly- or weakly-interact with hydration layer of solute, respectively (Gurney, 1953). Collins and Washbaugh proposed the “law of matching water affinity” which states that a kosmotropic cation forms an ion-pair only with a kosmotropic anion and a chaotropic cation forms ion-pair only with a chaotropic anion (Collins, 2004). The ion-pairs of the mixed types do not exist. A perfect match between the hydration enthalpies of oppositely charged ions inhibits them to interact with water. A mismatch in the hydration enthalpy allows the ions to dissolve in water and make ion-induced effects visible. A kosmotrope (water structure-maker) offers strong interaction to water molecules beyond its hydration layer than a chaotrope (water structure-breaker; Collins, 2006). However, these concepts were soon disproved by spectroscopic and thermodynamic considerations (Omta et al., 2003a; Batchelor et al., 2004; Funkner et al., 2012). According to these evidences, a salt ion only affects its first hydration layer and has no effect on the bulk water as expected.

For a classical ion, the kosmotropic and chaotropic character correlates with the relative viscosity by the Equation (1),

(1) $$\begin{array}{l} \frac{\eta }{\eta_{0}}=1+A\sqrt{c}+B.c \end{array}$$

where, η_0_ is the viscosity of solvent, η is the viscosity of salt solution at concentration *c, A* is the Falkenhagen coefficient that depends on the electrostatics of the system and are small and *B* is the Jones-Dole coefficient and represent the characteristic of ions (Collins, 1997). A kosmotropic ion interacts intensely with water layers, consequently increase the viscosity η and thus increase *B*, whereas the chaotropic ion due to their water-immiscible character possesses lower *B*. Except for some minor issues with anions, the Jones-Dole coefficient value is quite questionable for ionic liquid cations. The ionic liquid containing a longer alkyl chain on cation exhibits positive *B* values and hence considered as kosmotropic (more than eight carbon) (Zhao, 2006). The increase in the solution viscosity might be due to the hydrophobic hydration or because of the high viscosity of ionic liquids in water. Nevertheless, kosmotropic/chaotropic assignment of an ionic liquid cation is based on the *B* values, and their order in the Hofmeister series is different (Zhao, 2005, 2015; Yang, 2009). To avoid any complications, the Hofmeister series for anions used only for accounting the ion-induced effects.

Green noted that the protein solubility does not depend on the concentration of salt but also its ionic strength (*I*) in the solution (Green, 1932). For a solvent with protein solubility *S*_0_, which changes to *S* after addition of salt, depends on the ionic strength *I* by the Equation (2),

(2) $$\begin{array}{l} \text{log}\frac{s}{s_{0}}=\;\frac{1}{2}\;\frac{z_{1}.z_{2\sqrt{I}}}{1+A\sqrt{I}}-K_{s}.\;I \end{array}$$

where, *z*_1_ and *z*_2_ are the valences of the salt ions and *K*_*s*_ is salt coefficient and depends on the characteristic of salts. As *K*_*s*_ depends on the volume of ion, chaotropic ions noted to decrease the protein solubility (Salis and Ninham, 2014). Based on these factors, it is evident that the Hofmeister series do not depend on the ion but also its concentration and ionic strength in the solution.

### Ionic Liquid-Protein Interactions and Hofmeister Series

Similar to aqueous electrolytes, ionic liquids are constituted by oppositely charged ions held together by hydrogen-bonding and van der Waals interactions rather than strong electrostatic attractions as in case of electrolytes. The reduced charge density of ionic liquid ions weakens their interaction with the oppositely charged surfaces of proteins compared to the aqueous electrolytes. However, hydrophobic- and hydrogen bonding-interactions are noted to play anchoring role in the stabilizing protein structure. Hippel and Wong were the first to observe the Hofmeister ordering of tetraalkylammonium and guanidinium salts on the thermal stability of Ribonuclease A (RNase A), gelatin-collagen, DNA and precipitation of benzoic acid from aqueous state (Von Hippel and Wong, 1964). However, they did not overrule the possibility of the specific solute-ion interactions. Zhao et al. observed the Hofmeister ordering of ionic liquid ions during the hydrolysis of enantiomeric phenylalanine methyl ester catalyzed by *Bacillus licheniformis* protease in different aqueous ionic liquid solutions (Zhao et al., 2006). The kosmotropic anion and chaotropic cation were noted effective in enhancing the enzyme enantioselectivity. The decreasing order of anions: ${\text{PO}}_{4}^{3-}$ > citrate^3−^, CH_3_COO^−^, ${\text{EtSO}}_{4}^{-}$, CF_3_COO^−^ > Br^−^ > OTs^−^, ${\text{BF}}_{4}^{-}$ and for cations: [emim]^+^ > [bmim]^+^ > [hmim]^+^.

An ionic liquid solution with high kosmotropicity (which is given in terms of the difference of Jones-Dole B-coefficient of anion and cation) resulted in high enzyme enantioselectivity (Zhao et al., 2006). Mazzini et al. investigated the role of water, methanol, propylene carbonate (PC), dimethyl sulfoxide (DMSO) and formamide on the Hofmeister ordering of CH_3_COO^−^, F^−^, Cl^−^, Br^−^, I^−^, ${\text{ClO}}_{4}^{-}$, and SCN^−^ in the Size Exclusion Chromatography (SEC) of electrolyte solutions on *Sephadex*® G-10 and the investigation of the conformation of a polymer brush in the presence of the different electrolytes by Quartz Crystal Microbalance with Dissipation (QCM-D) (Mazzini et al., 2018). The fundamental Hofmeister ordering of ions maintained in the methanol and water (CH_3_COO^−^ > F^−^ > Cl^−^ > Br^−^ > I^−^ > ${\text{ClO}}_{4}^{-}$ > SCN^−^) whereas it reversed in case of DMSO and PC. There was no discrete ordering of ions in case of formamide (Mazzini et al., 2018). Constantinescu et al. measured the melting temperature (*T*_m_) of RNase A in presence of major cations such as 1-alkyl-3-methyl imidazolium ([Rmim]^+^), 1-alkyl-1-methylpyrrolidinium ([Rmpyrr]^+^), tetraalkylammonium ([R_4_N]^+^) and guanidinium ([Guan]^+^) and anions thiocyanate ([SCN]^−^), methylsulfate [MeOSO_3_]^−^), ethylsulfate ([EtOSO_3_]^−^), trifluoromethanesulfonate ([TfO]^−^), bis(tri-fluoromethanesulfonyl)imide ([Tf_2_N]^−^), and dicyanimide ([N(CN)_2_]^−^) (Constantinescu et al., 2007). The cation series in terms of decreasing *T*_m_ with Br^−^ and Cl^−^ as common anion is as,

$$\begin{array}{l} {\text{K}}^{+}>{\text{Na}}^{+}>{\left[{\text{C}}_{1,1,1,1}\text{N}\right]}^{+}>{\text{Li}}^{+}>{\left[{\text{C}}_{2,2,2,2}\text{N}\right]}^{+}\approx {\left[\text{emim}\right]}^{+}\\ >{\left[\text{bmpyrr}\right]}^{+}>{\left[\text{bmim}\right]}^{+}\approx {\left[{\text{C}}_{3,3,3,3}\text{N}\right]}^{+}>{\left[{\text{C}}_{6}\text{mim}\right]}^{+}\\ \approx \;{\left[{\text{C}}_{4,4,4,4}\text{N}\right]}^{+} \end{array}$$

**Figure 2:**
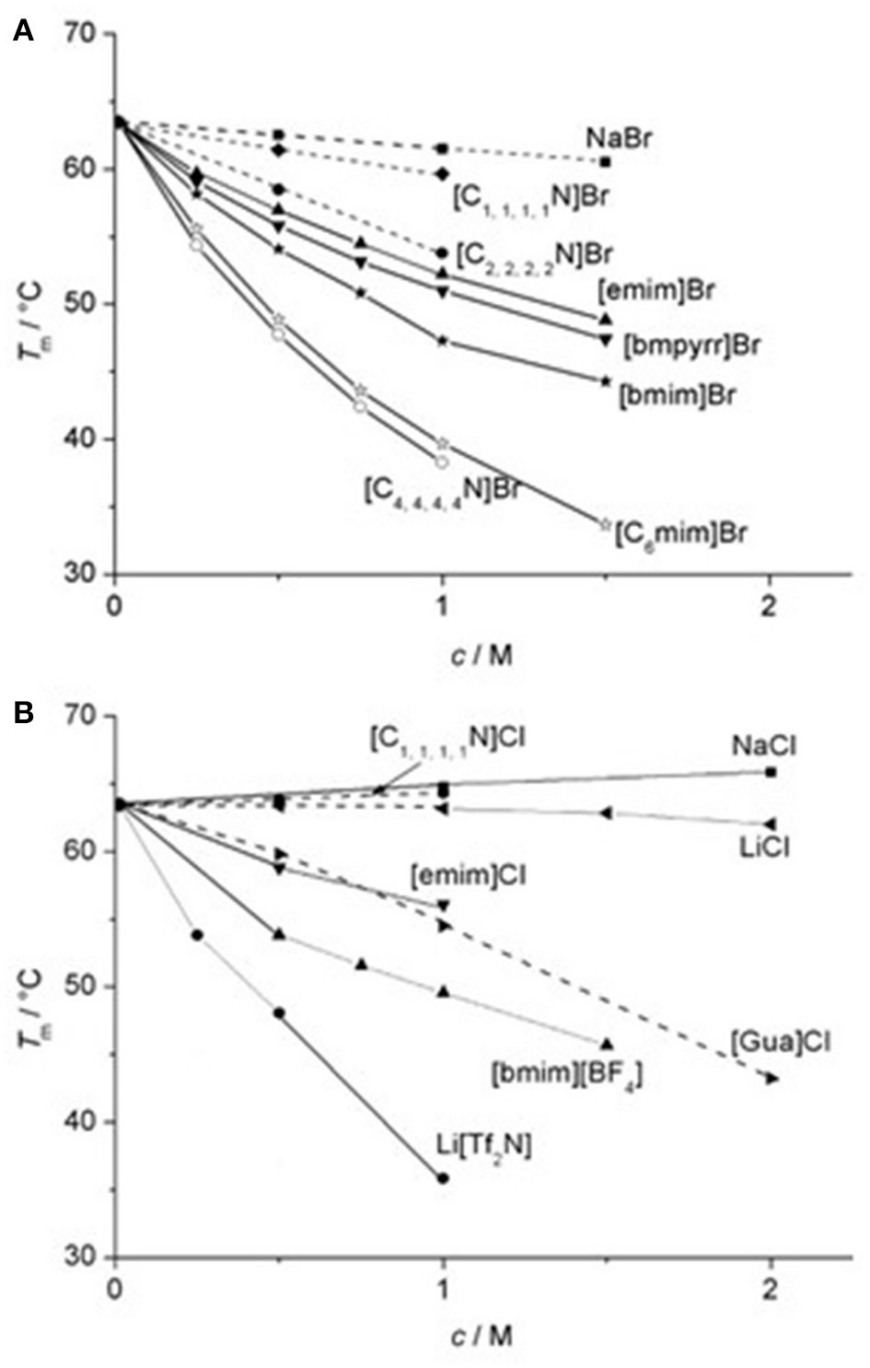
Transition temperatures *T*_m_ for the thermal denaturation of RNase A as a function of the concentration *c* of added ILs with **(A)** Br^−^ and **(B)** Cl^−^ as a common anion. Reprinted with permission from Constantinescu et al. (2007).

The thermal stabilization of RNase A by these cations was in the order of their hydrophobicity (Figure 2). The thermal stability data of lysozyme in presence of three imidazolium chloride-based ionic liquids fortifies this observation (Lange et al., 2005). The different ordering of structurally similar [emim]^+^ and [bmpyrr]^+^ ions was attributed to the charge delocalization in [emim]^+^. For a common cation [emim]^+^, the order of anions in terms of decreasing *T*_m_ values is as,

$$\begin{array}{l} {\left[\text{S}{\text{O}}_{4}\right]}^{2-}>{\left[{\text{HPO}}_{4}\right]}^{2-}>{\text{Cl}}^{-}>{\left[{\text{EtOSO}}_{3}\right]}^{-}>{\left[{\text{BF}}_{4}\right]}^{-}\approx {\text{Br}}^{-}\\ >{\left[\text{MeOS}{\text{O}}_{3}\right]}^{-}>{\left[\text{TfO}\right]}^{-}>{\left[\text{SCN}\right]}^{-}\approx {\left[\text{N}{\left(\text{CN}\right)}_{2}\right]}^{-}>\;{\left[{\text{Tf}}_{2}\text{N}\right]}^{-} \end{array}$$

**Figure 3:**
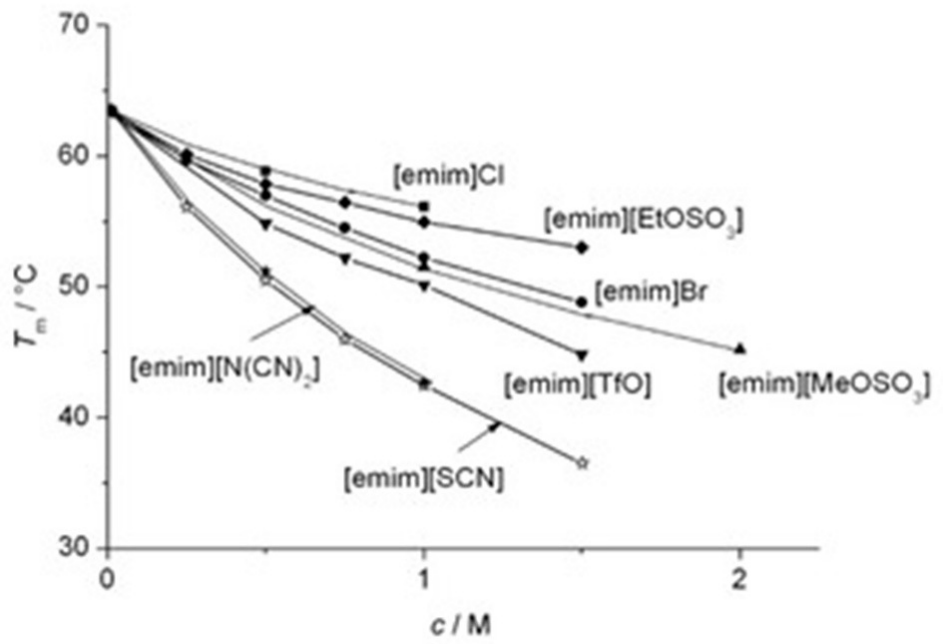
Transition temperature *T*_m_ for the thermal denaturation of RNase A as a function of the concentration *c* of added ILs with [emim]^+^ as a common cation. Reprinted with permission from Constantinescu et al. (2007).

The series suggests that weak hydration and high hydrophobicity tends to decrease the *T*_m_ more (Figure 3). However, this should not be taken as a rule of thumb in arranging the anions on the basis of their effectiveness in stabilizing/destabilizing the RNase A since all these anions belong to different homologous series. Even for the chemically-related anions, the order of hydrophobicity is not valid as observed from their effect on *T*_m_ (Zhao, 2006). The reverse ordering at various places in the cation and anion series suggests that the simple hydration theory cannot be applied for the complex ions and thus, this assumption is not valid in case of ionic liquids. Zhao also noted that enzyme activity in aqueous ionic liquids seems to follow the Hofmeister series as ions remain separated but predicting their activity in absence of water is not a straightforward affair (Zhao, 2005). Lau et al. had similar observation upon testing the influence of pure ionic liquids on the activity of *Candida antarctica* Lipase B (CaL B) (Lau et al., 2004). They noticed that the concept of kosmotropicity/chaotropicity and Jones-Dole B-coefficient cannot be applied to account for the influence of ionic liquids over the activity of CaL B. Even for the similar [NTf_2_]^−^ anion counter cation such as Li^+^ destabilizing behavior was noted for Cal B while a stabilizing behavior was evident with [emim]^+^ (de Diego et al., 2005; Constantinescu et al., 2007). These observations suggest that binding of ionic liquids to protein surface depends on the charge and hydrophobicity and thus indicates specific ion-protein interactions.

### Beyond Hofmeister: Specific Ion-Solute Interactions

The reports by Omta, Jungwirth, and Funkner on the protein interaction in aqueous electrolytes strongly suggested that the ions interaction toward the protein backbone is driven by specific ion-protein interactions and not necessarily due to the perturbation of the bound water to the protein (Omta et al., 2003a; Batchelor et al., 2004; Heyda et al., 2010; Algaer and van der Vegt, 2011; Funkner et al., 2012; Jungwirth and Cremer, 2014). These observations were confirmed as valid in several studies utilizing ionic liquids. Kumar et al. compared the stabilizing power of SCN^−^, ${\text{SO}}_{4}^{2-}$, ${\text{HSO}}_{4}^{-}$ Cl^−^, Br^−^, I^−^, and CH_3_COO^−^ for ionic liquids with 1-butyl-3-methylimidaolium ([bmim]^+^) cation and for inorganic salt with sodium ion on α-chymotrypsin (CT) (Kumar et al., 2014). The comparison showed that the SCN^−^, ${\text{SO}}_{4}^{2-}$, ${\text{HSO}}_{4}^{-}$ Cl^−^, Br^−^, I^−^, and CH_3_COO^−^ of sodium salt have a destabilizing effect whereas the Cl^−^, Br^−^, and CH_3_COO^−^ with [bmim]^+^ have a stabilizing effect on the CT. The arrangement of these anions in the stabilizing/destabilizing order of CT does not necessarily follow the Hofmeister ordering and depends a lot on the counter cation and nature of the protein in question. Similarly, the influence of ionic liquids on the stability of collagen and activity of laccase do not follow the Hofmeister ordering of anions and indicate specific anion-protein binding (Sun et al., 2017; Tarannum et al., 2019). Reid et al. investigated various theories predicting the behavior of ionic liquid in water by statistical thermodynamic. The structure of water-IL mixture was water dependent (Reid et al., 2015). At a lower concentration, water binds strongly with ion whereas at higher concentrations, water molecules aggregate themselves and interact less with ionic liquid. Kobayashi et al. noted similar behavior of water at the water-ionic liquid interface in their study of ionic liquid-water interaction study by molecular dynamics and sum frequency generation (SFG) spectroscopy, at different concentrations of water (Kobayashi et al., 2017, 2019). Bui-Le et al. used a set of analytical techniques such as circular dichroism (CD), fluorescence, UV-visible, NMR and small-angle X-ray scattering to probe the protein [green fluorescent protein (GFP)] interaction with imidazolium and pyrrolidinium as cation and chloride, acetate and triflate as complementary anions (Bui-Le et al., 2020). The site-specific protein-ionic liquid interactions through various analytical methods exhibited triflate as the stabilizing anion while to chloride and acetate as the destabilizing anions. Singh et al. studied the thermal unfolding of lysozyme in 1-ethyl-3-methylimidazolium ethylsulphate ([emim][EtSO_4_]) and 1-ethyl-3-methylimidazolium diethylphosphate ([emim][Et_2_PO_4_]) both experimentally and theoretically (Singh et al., 2020). The destabilizing power of [emim][EtSO_4_] and [emim][Et_2_PO_4_] was higher than that of a strong denaturant as observed from lowering in the *T*_m_. Among ionic liquids, [emim][Et_2_PO_4_] was more destabilizing for lysozyme and required lower energy, as is evident from Δ*H*, than [emim][EtSO_4_]. The fluorescence study showed that both ionic liquids interact with the tryptophan residue of lysozyme. MD simulation revealed that cation interacts in a “local-manner” while anion in a “global-manner” due to the negative charge of the lysozyme. The [Et_2_PO_4_]^−^ was found to have closer first coordination shell and stronger coulombic interaction with lysozyme than [EtSO_4_]^−^.

Besides the experimental work, several theoretical studies on the ionic liquid-protein interactions indicated a trend pointing toward the specific ion-solute interactions. Lesch et al. studied the influence of aqueous [emim][CH_3_COO] on the stability of β-hairpin peptide using atomistic MD simulation and Kirkwood-Buff theory. The simulation work suggested that the cation ([emim]^+^) binds with both folded and unfolded peptide but anion ([CH_3_COO]^−^) binds only with the unfolded peptide (Lesch et al., 2015). In an another work Diddens et al. compared the behavior of different anions ([BF_4_]^−^, [CH_3_COO]^−^, and Cl^−^) with the same cation on the ionic l iquid-protein using the atomistic MD simulation and Kirkwood-Buff theory (Diddens et al., 2017). The simulation outcome suggests that the larger anion interacts with protein surface more strongly, followed by dehydration, than the smaller anion and the interaction was enthalpic in nature. Jaegar and Pfaendtner studied the stability of human serum albumin (HSA) in 1-butyl-3-methylimidazolium tetrafluoroborate ([bmim][BF_4_]) and choline dihydrogenphosphate ([chol][dhp]) by MD simulations and enhanced sampling techniques (Jaeger and Pfaendtner, 2016). The RMSD and RMSF calculations indicated that at higher ionic liquid concentrations, the protein adopts structure similar to their crystallographic structure. The structure of HSA in 20% [chol][dhp] is similar to that in water and thus it is unlikely that [chol][dhp] destabilize the HSA in its pure state. Burney et al. investigated the modification of the enzyme surface charge for *Candida rugosa* lipase and *Bos taurus* α-chymotrypsin in aqueous 1-butyl-3-methylimidazolium chloride ([bmim]Cl) and 1-ethyl-3-methylimidazolium ethylsulphate ([emim][EtSO_4_]) using MD simulations (Burney et al., 2015). The calculated solvent charge density indicated that for both enzymes in ionic liquids changed their positively charge surface to the negatively charged one upon an increase in the solvent concentration near the enzyme surface. The radial distribution of ionic liquid components with respect to enzyme reveals the decreased interaction of anion with the modified surface and more to the cation. Ghanta et al. showed in their MD simulation on α-lactalbumin in aqueous [bmim][BF_4_] the conformational changes in the protein as well as the distribution of water and ionic liquid around it (Ghanta et al., 2020). The calculations revealed an enhanced rigidity of protein due to the rearrangement of protein-water hydrogen bond and the formation of protein-ionic liquid hydrogen bond. The formation of greater number of salt bridges in presence of ionic liquid also account for the enhanced rigidity. Jaegar et al., compared the aqueous ionic liquid ([bmim][CH_3_COO]) tolerance on cellulases from *Trichoderma viride, Thermogata maritima*, and *Pyrococcus horikoshii* at different temperatures and different concentrations of ionic liquid in water (Jaeger et al., 2014). The simulation results indicated dissimilar effects of the ionic liquid on enzyme deactivation. The most negatively charged enzyme was least destabilized and had similar behavior in water and binary mixtures of ionic liquids. A summary of interactions between ionic liquid moieties and protein and DNA surfaces are given in Table 2. Majority of research about the protein-ionic liquid interactions do not account the solvation properties of ionic liquids. A correlation between the solvation parameters of ionic liquids and their influence on the fate of protein can be of paramount value in designing the potential optimum ionic liquids. Tomlinson et al. studied the solubility of corn protein zein in aprotic ionic liquids, namely, 1-butyl-3-methylimidazolium acetate ([bmim][CH_3_COO]), 1-ethyl-3-methylimidazolium acetate ([emim][CH_3_COO]), and 1-butyl-3-methylimidazolium dicyanamide ([bmim][N(CN)_2_]) and protic ionic liquids containing 1-methylimidazolium ([Hmim]^+^) cation with [HSO_4_]^−^, [CH_3_COO]^−^, and [HCOO]^−^ as anions (Tomlinson et al., 2014). Zein dissolved in all ionic liquids except [Hmim][HSO_4_]. While comparing the zein solubility among ionic liquids, [bmim][N(CN)_2_] exhibited the highest solubility owing to the denaturing action of dicyanamide anion. The other aprotic ionic liquids [emim][CH_3_COO] and [bmim][CH_3_COO] showed similar solubilization properties despite their different solvation properties. The importance of solvation properties [*E*_T_(30), α, β, and π^*^] is however more evident in protic ionic liquids. At 60°C, zein had a maximum solubility in [Hmim][CH_3_COO] (26.2 wt%) followed by [Hmim][HCOO] (12.3 wt%) whereas the lowest solubility was found in [Hmim][HSO_4_] (< 1 wt%). The decreasing order of zein solubility was in line with the increasing order of polarity [*E*_T_(30)], indicating that least polar protic ionic liquid are required for higher solubility. Single and multivariate regression using polarity parameters and molar volume on zein's solubility in ionic liquids suggested that a small molecular size and low *E*_T_(30), α, β, and π^*^ are required to attain maximum solubility of zein in an ionic liquid. The higher solubility of zein in [bmim][CH_3_COO] than in [emim][CH_3_COO] is due to the lower π^*^ for former than latter. The lower correlation coefficient for α suggests that acidity has lower impact on the zein's solubility. π^*^ is noted as the important parameter and at higher temperature its highly responsible for the non-polar character to achieve higher solubility. Despite the effective correlation between zein's solubility and polarity parameters, more studies are needed before reaching any definite conclusions.

**Table 2:** Summary of interactions between ionic liquids and sites of proteins and DNA.

**Functional group on ionic liquids**	**Interaction with protein/DNA**	**References**
Alkyl chain on cation	Hydrophobic sites on the proteins [human serum albumin (HSA), bovine serum albumin (BSA), and lipase A] and major grooves of DNA	Chandran et al., 2012; Singh et al., 2012; Nordwald et al., 2014
Electron-donor substituents on cation	Disulphide linkages on protein	Liu et al., 2015
Nucleophilicity of anion	Disulphide linkages, positively charged surface on protein [*Candida antarctica* lipase B (CAL-B)], cytochrome c (Cyt. C), DNA bases (adenine, guanine, and cytosine), and G-quadruplexes	Fujita et al., 2005; Cardoso and Micaelo, 2011; Klähn et al., 2011a; Fujita and Ohno, 2012; Liu et al., 2015
Electrophilicity of cation	Phosphate groups on DNA strands and negatively charged surfaces on protein	Klähn et al., 2011a; Sarkar et al., 2020;
Hydroxyl group (-OH) on choline cation	Hydrogen bonding with carbonyl (C=O), imine (-N-H) and hydroxyl (OH) group on protein (e.g., collagen protein)	Tarannum et al., 2018
Anionic aggregates (e.g., octylsulphate, [OS])	Acidic sites on protein [e.g., lysozyme (tryptophan, arginine, *etc*.)] and hydrophobic sites on BSA	Mandal et al., 2015
Cationic aggregates (e.g., 1-methyl-3-octylimidazolium cation, [Omim]^+^)	Hydrophobic and electrostatic interactions with carboxyl and amine functionality on BSA	Singh et al., 2012
Hydrophobic cation (e.g., cyclic alkylmethylguanidinium cation [RR'GUA]^+^)	Hydrophobic core of protein [e.g., CAL-B, Ribonuclease A (RNase A), and α-helical protein (Im7)]	Constantinescu et al., 2007; Klähn et al., 2011b; Figueiredo et al., 2013
Highly polar ionic liquid (e.g., [bmim][NO_3_] and [OHemim][BF_4_])	Polar surfaces of protein CAL-B	Klähn et al., 2011b
Hydrophobic anion (e.g., [N(CN)_2_]^−^, [NTf_2_]^−^)	Hydrophobic core of RNase A	Constantinescu et al., 2007
Acidic proton on cation	Negatively charged residues on protein and base-pair of DNA	Sarkar et al., 2020
π-electrons on cation	NH—π interactions with DNA bases	Cardoso and Micaelo, 2011

### Influence of Water on Ionic Liquid-Protein Interactions

An aqueous solution of ionic liquids is used in the storage of biomolecules to alleviate the solubility problem and to maintain their structural features intact. Aqueous ionic liquids have different properties than neat ionic liquids but resemble closely to dilute electrolytes solution. The amphiphilic character of ionic liquids makes them heterogeneous, at the molecular level, that further increases with the size of alkyl chain on cation (Hayes et al., 2015; Bruce et al., 2017). Addition of water alters the heterogeneity of ionic liquids (Jiang et al., 2007). Blesic et al. showed that imidazolium chloride-based ionic liquids with alkyl chain greater than octyl undergoes self-aggregation whereas an ionic liquid with shorter chains (butyl to hexyl) do not show such behavior (Blesic et al., 2007). Liu et al., observed rod-like micelle in imidazolium-based ionic liquids whereas Vincent-Luna et al. noticed that critical micelle concentration required for micelle formation was lowered with an increase in the anisotropy in the imidazolium-based ionic liquids (Liu et al., 2015; Vicent-Luna et al., 2017). Cammarata et al. showed that water binds with ionic liquid anion in 1:2 ratio and the water content of ionic liquids increases with the basicity of anion (Cammarata et al., 2001). Simulation studies also indicate that water cluster size in ionic liquids are independent of the size of alkyl chain length on cation although the hydrophobicity of anion is crucial in deciding the miscibility with water (Méndez-Morales et al., 2011). The orientation of water molecules at the surface of hydrophilic and hydrophobic ionic liquids are different (Anthony et al., 2001; Rivera-Rubero and Baldelli, 2004). In hydrophilic ionic liquids, water molecules experience stable dipole-dipole and hydrogen bonding interactions and remain in solvated state whereas water molecules undergo surface orientation at gas-liquid interface in case of the hydrophobic ionic liquids (Anthony et al., 2001; Rivera-Rubero and Baldelli, 2004). Addition of water in ionic liquids also affects the dynamics of ions as observed in many studies (Araque et al., 2015; Sharma and Ghorai, 2016).

The consequence of water addition to the protein-ionic liquid interactions cannot be generalized based on its influence over the ionic liquids. In reality, both stabilizing and destabilizing effects of water on the protein-ionic liquid interactions were observed. Micaêlo and Soares study on a model protein in presence of [bmim][PF_6_] suggested that a higher fraction of water molecules resides in the solvation shell of protein and hence higher stability was observed compared to that in case of [bmim][NO_3_] (Micaêlo and Soares, 2008). Similarly, a decrease in the hydrodynamic radius of lysozyme was observed upon addition of 1-propyl-3-methylimidazolium bromide ([pmim]Br) in aqueous lysozyme (Ghosh et al., 2015). Conversely, higher activity of α-chymotrypsin was observed in [bmim][NTf_2_] compared to the organic solvents at low water concentration (Eckstein et al., 2002). These contradictory observations about the role of water on protein stability in presence of ionic liquids indicate that the fate of the protein depends on its direct interaction with ionic liquids and do not depend on the interaction of water on the ionic liquids as assumed in the simple hydration theory. However, addition of water alters the stabilizing/destabilizing role of ionic liquids toward proteins and enzymes. As noted by Constatinescu et al., a strong interaction between cation and anion in ionic liquids might reduce the destabilizing effect of cation or anion of ionic liquids (Constantinescu et al., 2010). The role of water in protein stabilization in presence of ionic liquid is also controversial. In some cases, higher concentrations of ionic liquids were needed for the long-term stability. However, a change in the tertiary structure of protein was also reported at higher concentrations of ionic liquids (Byrne et al., 2007; Bihari et al., 2010). Singh et al. showed that long-chain imidazolium-based ionic liquids stabilize the horse heart cytochrome c (h-cyt-c) for a long term and at very low concentrations (1mM) (Singh et al., 2018). However, the presence of water in ionic liquid-protein mixtures is shown to have a distinct effect on the structural features. Proteins with the radius of gyration below 20 Å interact with water and remain in solvated state whereas proteins having radius of gyration above 20 Å do not react with water and undergo protein-protein interactions and result in larger aggregates (Takekiyo et al., 2014). The review articles by Smiatek (2017) and Saha and Mukherjee (2018) present more evidence about the inconclusive role of water on the ionic liquid-protein interactions.

## Implications of Ionic Liquid-Protein Interactions

This section accounts for various applications of ionic liquid-protein interactions in protein chemistry. Firstly, the effect of various features of ionic liquid systems on the protein solubility is discussed. Secondly, use of ionic liquids in protein separation is discussed by making use of the ionic liquids-based aqueous biphasic systems (ABSs) and electrophoretic processes. Lastly, protein stability in ionic liquids-based systems is also discussed by using enthalpy-entropy compensation, role of the Hofmeister series on stabilizing protein and thermodynamics of the Hofmeister effects.

### Protein Solubility

Though protein is fairly soluble in ionic liquids, however, an aqueous mixture of ionic liquids is used for low cost and high solubility of biomolecules. A mixture of ionic liquid and water better interacts with the polar, non-polar, or amphiphilic surfaces of proteins because of the hydrophilic, hydrophobic, and amphiphilic characters than the neat ionic liquids. These interactions cause either the precipitation or stabilization of proteins. In ionic liquids, the anion remains in the hydrated state more than the cation and hence the concentration of cation at the surface of the protein remains higher as obtained by various simulation results (Haberler and Steinhauser, 2011; Haberler et al., 2011, 2012; Klähn et al., 2011a,b; Lesch et al., 2015). The cation tethered with a long alkyl chain exhibits amphiphilic character; the cation being polar and alkyl moiety acts as a non-polar region (Zhang and Cremer, 2006). Because of the non-polar character, the alkyl chain orients toward the non-polar surface of proteins and the cation remains at the polar surface (Lim et al., 2014). According to the “iceberg model,” water molecules surround the alkyl chain and consequently lower the entropy of water molecules, and result in the hydrophobic hydration (Zangi, 2010). The ionic liquid cation competes with the anion toward the polar surface of proteins. The negatively charged amino acid moiety such as glutamic- and aspartic-acid exclusively attract the cation but as water molecules also remain in the equilibrium toward the site, due to hydrogen bond formation, the extent of attraction remains lower (Haberler and Steinhauser, 2011; Klähn et al., 2011a). As compared to cation, high charge density anion strongly interacts with amino acid moiety *per se* histidine, arginine, and lysine by the coulombic interactions that decide the fate of protein in aqueous ionic liquid solutions (Seduraman et al., 2012). Overall, the ionic components interact stronger at the protein surface than water hence replacing the bound water from the protein surface and hence resulting in coagulation or crystallization of a protein (Zaks and Klibanov, 1988). On the contrary, hydrophobic molecules stabilize the protein surface owing to their tendency of not interacting with water (Laszlo and Compton, 2002; Dang et al., 2013).

Additionally, the ionic liquid concentration also affects the protein solubility as the ion interacts strongly with the surrounding water hydrogen-bonding network of protein. At moderate ion concentrations up to 1 mol.l^−1^, the ion breaks the hydrogen bonding network of protein and increases its solubility by the phenomenon called “salting-in” (Thomas and Elcock, 2007). The strong chaotropic anion promotes the salting-in while a reverse effect noticed with the kosmotropic ions referred to as “salting-out.” The energetics of salting-in might be entropic/enthalpic, but in the case of salting-out, it is purely enthalpic (Zangi et al., 2007). Later, several MD simulation results indicated that the salting-in and salting-out not rely on the ion concentration but also depends on the specific ion-protein interactions because of the non-homogenous charge distribution, hydrophobicity/hydrophilicity, and difference in the functional structures of the protein (Zangi, 2010; Schwierz et al., 2013). The Hofmeister ordering was intact in case of the negatively charged ions but completely reversed for cations (Schwierz et al., 2016).

The control of protein precipitation and subsequent crystallization by tuning the salt concentration in protein solution is necessary for the structure determination of protein by X-ray diffraction (Judge et al., 2009). Ionic liquids, because of large availability and recyclability considered as a better co-solvent, and initial results established them as an excellent crystallizing agent for protein. The other benefits using ionic liquids in protein crystallization were less polymorphism and improved tolerance to concomitant impurities in crystals. The method was used to obtain the highly pure crystals of protein at the required salt concentrations. In 1999, Garlitz et al. reported lysozyme crystallization using ethylammonium nitrate (Garlitz et al., 1999). Later, Judge et al. noticed that proteins such as lysozyme, catalase, myoglobin, trypsin, glucose, and isomerase grow bigger crystals with ionic liquids as co-solvents and provide better resolution in x-ray crystallography (Judge et al., 2009). Kowacz et al. observed a reduction in nucleation density and improved crystal quality at higher ionic liquid concentration (Muldoon et al., 2012). In a comparison, choline chloride was noted less efficient than imidazolium-based ionic liquids in protein crystallization. The efficacy of imidazolium-based ionic liquids in crystallization was noted to increase with the length of the alkyl chain upon cation. Green observed the importance of electrostatic interaction at low ion concentrations (Ajaj, 2010). The electrostatic force is first screened by the like-charged biomolecular region that helps in protein crystallization (Muldoon et al., 2012).

### Protein Separations

The efficiency and viability of any biotechnological process are in its efficiency to downstream processing to maintain the purity and quality, as it costs 60–90% of the overall process (Kula et al., 1982). The metabolites and bioproducts separation depends on the delicate change in pH, temperature, osmotic pressure, ionic strength, and surface charges; therefore, the techniques/methods utilized for the separation must be reliable and compatible with the bioproducts (Banik et al., 2003; Neves et al., 2009). Conventional methods used for the bioproducts separation from biotechnological processes are cost-ineffective and result in poor yields (Silva and Franco, 2000). In pure ionic liquids, most proteins get dissolved but not homogeneously dispersed. Besides, they can also denature the protein. In place of pure ionic liquids, an aqueous-biphasic solution (ABS) of the ionic liquid has emerged as an effective tool in biomolecular separation. An aqueous solution of ionic liquid with a salt solution can be used to create phase separation between aqueous ionic liquid and aqueous salt solution and therefore allows easier separation of bioproducts than conventional methods. Although hydrophobic ionic liquids form a biphasic solution with water but their high cost and protein denaturation ability render them unsuitable for biomolecule separations.

#### Aqueous-Biphasic Solution (ABS)

An ABS is formed when two mutually immiscible but water-soluble polymer/polymer, polymer/salt, and salt/salt systems are employed together. For the first time, Gutowaski et al. exhibited that 1-butyl-3-methylimidazolium chloride ([bmim]Cl), a hydrophilic ionic liquid, undergoes aqueous biphasic separation (ABS formation) upon mixing with the concentrated solution of K_3_PO_4_, a water-structuring salt (Gutowski et al., 2003). The upper layer remains rich in ionic liquid and lower layer rich in K_3_PO_4_. These new ABSs were first supposed to be used in the recycling of hydrophilic ionic liquids and metathesis in the formation of new ionic liquids but were later used in the extraction and separation of biomolecules and transition metal elements. The first use of ionic liquid-based ABS was noted by He et al. where they coupled [bmim]Cl + K_2_HPO_4_ + water-based ABS with reversed-phase high-performance liquid chromatography (RP-HPLC) for concentration and analysis of testosterone and epitestosterone (He et al., 2005). Later, Coutinho et al. extensively studied the utilization of ionic liquid-based ternary ABSs in the separation of the amino acid L-tryptophan with the major emphasis on the role of structural features of ionic liquids. They observed that separation of L-tryptophan increases with the length of alkyl chain on the cation because of elevated hydrophobicity. Further, the cation hydrophilicity increases the biphasic separation whereas the tethering of polar group such as hydroxyl, benzylic, and allylic increases the interaction with water and hence decreases the phase separation (Gutowski et al., 2003; Neves et al., 2009). The role of the anion in biphasic separation and extraction of L-tryptophan was studied by Ventura et al. by employing 1-ethyl-3-methylimidazolium- ([emim]) and 1-butyl-3-methylimidazolium ([bmim])-based ionic liquids with chloride, bromide, acetate, hydrogensulfate, methanesulfonate, methylsulfate, ethylsulfate, trifluomethanesulfonate, trifluoroacetate, and dicyanamide as the anion (Ventura et al., 2009). The results indicated that the extraction ability of prepared ABSs depends inversely on the hydrogen bond donor basicity of anion with water. Dreyer et al. pointed out that extraction of protein in ionic liquid (Ammoeng^TM^ 110)-based two-phase ABS depends on the electrostatic interactions between the positively charged cation and charged surface of the protein (Dreyer et al., 2009). Rather than polymer-based ABS, where hydrophobicity is a deciding parameter, ionic liquid-based ABSs remain unaffected of the hydrophobicity or surface area. It was concluded that the protein separation using ionic liquid -based ABSs does not simply depend on the biphasic separation but, in reality, is a complex phenomenon. Pei et al. used aqueous K_2_HPO_4_ + [C_n_mim]Br] (where *n* = 4, 6, and 8)-based ABSs for the extraction of bovine serum albumin (BSA), cytochrome c, trypsin, and γ-globulin at different temperatures and pH conditions to achieve 75–100% extraction efficiency (Pei et al., 2009). The extraction efficiency was noted to change with the temperature and alkyl chain length on the cation. A small change in the extraction efficiency was observed with the pH change for cytochrome c due to the change of isoelectric point and hence electrostatic interaction between cytochrome c and ionic liquid cation. The thermodynamic study confirmed that increased extraction efficiency with temperature is owing to the endothermic nature of the process in ABSs keeping in mind that the temperature change should be below the denaturation temperature of protein. The standard Gibbs free energy for the extraction process at a given temperature can be correlated by the van't Hoff Equation (3)

(3) $$\begin{array}{l} \Delta G_{o}^{T}=\;-RTlnK \end{array}$$

where, *K* is the partition coefficient of protein between two phases at temperature *T* (*K*) at gas constant *R*.

The standard enthalpy (Δ*H*^*o*^)- and standard entropy (Δ*S*^*o*^)- change associated with protein partitioning can be obtained by Equation (4)

(4) $$\begin{array}{l} lnK=\;-\frac{\Delta H^{o}}{RT}+\frac{\Delta S^{o}}{R} \end{array}$$

The Δ*H*^*o*^ and Δ*S*^*o*^ can be obtained from slope and intercept by plotting $lnK\;vs\;\frac{1}{T}$.

The values of both enthalpy (Δ*H*^*o*^) and entropy (Δ*S*^*o*^) are positive but $\Delta G_{o}^{T}\;$is negative. The *T*Δ*S*^*o*^ > Δ*H*^*o*^ indicated that the separation process is entropically favored in studied ABSs. The UV-vis- and IR-spectra showed that the protein structure remains intact even after the extraction with ABSs.

#### Capillary Electrophoresis (CE) and Micro-Capillary Electrophoresis (μCE)

Apart from the use of biphasic solution in protein separation, a capillary electrophoretic method is also used in protein separation and detection. In this method, the ionic liquid is used as a running electrolyte solution that causes a reversal of surface charge on the column wall (Jiang et al., 2003). By employing 1-ethyl-3-methylimidazolium tetrafluoroborate ([emim][BF_4_]) as a dynamic coating, the absorption of the protein was reduced at the wall of column and paved way for the separation and detection of proteins such as lysozyme, cytochrome c, trypsinogen, and α-chymotrypsinogen A. The separation was observed to facilitate upon increasing the length of alkyl chain on cation. Li et al. used 3% (v/v) ionic liquid solution in capillary electrophoresis for the detection of model protein avidin (Li et al., 2007). Protein detection was bettered by increasing ionic liquid concentration. Corradini et al. in their study showed that flushing a solution of 1-alkyl-3-methylimidazolium-based ionic liquid into a bare-fused silica capillary produces a non-covalent layer on the inner surface of coating that affects the untoward interaction of the protein with the capillary wall even if ionic liquid not present with the electrolyte (BGE) used for separation (Corradini et al., 2009). This effect was attributed to the adsorption of ionic liquid on the silica wall, which subsequently reduces the electrophoretic behavior of basic proteins up to different extent depending on ionic liquid. Xu and Wang published a detailed account on the use of ionic liquids in the improvement of capillary and microchip electrophoresis for the separation and detection of analytes such as phenols and aromatic acids, metal ions, medicines, enantiomers, biological materials, etc. (Xu and Wang, 2009).

### Protein Stability

Protein is a heterogeneous class of biomolecule whose stability depends on the preservation of its functional secondary structure, which is α-helical, β-sheet and coil region. The functional structure of protein is a complex phenomenon and depends on the hydrogen bonding, disulfide (S-S) bond, hydrophobic, and intramolecular interactions. To maintain its functionality, a protein requires a specific solvent, pH, co-solvent and temperature. Any change in these conditions alters the functionality of the secondary structure of the protein. Apart from these conditions, viscosity of solvent also affects the dynamics of solvent and protein (van Rantwijk and Sheldon, 2007). At higher viscosity, the diffusion of protein is slow with the insignificant change in the catalytic ability. The hydrolysis of some [PF_6_]- and [BF_4_]-based ionic liquids form HF and alters the protein functionality (van Rantwijk and Sheldon, 2007). The enthalpy-entropy compensation and the relevance of the Hofmeister series of ions on protein stabilization is discussed below.

#### Enthalpy-Entropy Compensation

The marginal/low stability of protein is represented in terms of Gibb's free energy of unfolding (Δ*G*_u_) using enthalpic (Δ*H*_u_) and entropic (Δ*S*_u_) contributions (Equation 5).

(5) $$\begin{array}{l} \Delta G_{\text{u}}=\;\Delta H_{\text{u}}-T\Delta S_{\text{u}} \end{array}$$

The Δ*G*_u_ accounts for the thermodynamic stability of a protein from native to the unfolded state and should be positive for a stable conformation (Lumry and Rajender, 1970). However, a low Δ*G*_u_ indicates that protein stabilization largely depends on the higher enthalpic and entropic contributions that are also called “enthalpy-entropy” compensation, whose value can be manipulated by the addition of co-solute in the system (Senske et al., 2014). In general, the co-solute which can be excluded from the protein structure favors folded state while those binds to the protein surface shift the equilibrium toward the unfolded state of the protein. Macromolecular crowders, like polyethylene glycol and dextran, are noted to be excluded co-solute. They protect protein either due to the entropic excluded volume effect or enthalpic effects (Zhou et al., 2008; Sapir and Harries, 2015). Urea and guanidinium salts are chemical denaturant and destabilize the proteins by direct interactions that reduce Δ*H*_u_ (Benton et al., 2012; Senske et al., 2014).

Similar to the inorganic/organic materials, ionic liquids may also cause a subtle change between the Δ*H*_u_ and Δ*S*_u_ when used as a co-solute and hence shift the equilibrium between the folded to unfolded states. For example, choline dihydrogen phosphate stabilizes cytochrome *c* and lysozymes for months (Fujita et al., 2006, 2007; Vrikkis et al., 2009). The stabilization mechanism involves the protection of the hydrophobic part of protein by the cation of ionic liquids (Summers and Flowers, 2000; Kumar and Venkatesu, 2013, 2014).

Apart from stabilization, ionic liquids have also been employed in accelerating the protein activity. The mechanism may involve either the chemical modification or stabilization or immobilization of the protein surface for chemical reactions (Zhao, 2006; van Rantwijk and Sheldon, 2007). However, the activity of lipase-catalyzed transesterification of methyl methacrylate and the polytransesterification of divinyl adipate and 1,4-butanediol in hydrophilic ionic liquids exhibited no reaction despite the high activity of the enzyme in 1-butyl-3-methylimidazolium hexafluorophosphate ([bmim][PF_6_]) (Kaar et al., 2003). The ineffectiveness of lipase is tested by adsorption, PEG-modification, and covalent immobilization in polyurethane foams in the hydrophilic ionic liquids.

#### Protein Stability and Hofmeister Series

Based on the change in the melting temperature (*T*_*m*_) of ribonuclease A (RNase A) in various ionic liquids and inorganic salts, Weingärtner observed the trend of ions similar to the Hofmeister series (Weingärtner et al., 2012). The series shows that both the stabilizing and destabilizing effects to the native structure of protein depends on the choice of ions and hence to the extent of intermolecular interactions between them (Constantinescu et al., 2007, 2010).

$$\begin{array}{l} {\text{K}}^{+}>\text{N}{\text{a}}^{+}>\left[\text{M}{\text{e}}_{4}{\text{N}}^{+}\right]∥\text{L}{\text{i}}^{+}>[\text{cho}{\text{l}}^{+}]>[\text{E}{\text{t}}_{4}{\text{N}}^{+}]\\ ≃[{\text{C}}_{2}\text{mi}{\text{m}}^{+}]>\text{gu}{\text{a}}^{+}>[{\text{C}}_{4}{\text{C}}_{1}{\text{p}}_{\text{y}}^{+}]>[{\text{C}}_{4}\text{mi}{\text{m}}^{+}]≃[\underset{4}{\text{Pr}}{\text{N}}^{+}]\\ >[{\text{C}}_{6}\text{mi}{\text{m}}^{+}]≃[\text{B}{\text{u}}_{4}{\text{N}}^{+}] \end{array}$$

where, *chol*^+^ and *gua*^+^ are acronyms for choline and guanidinium, respectively.

$$\begin{array}{l} \text{S}{\text{O}}_{4}^{2-}>{\text{H}}_{2}\text{P}{\text{O}}_{4}^{-}>\text{C}{\text{H}}_{3}\text{CO}{\text{O}}^{-}>{\text{F}}^{-}>\text{C}{\text{l}}^{-}∥{\text{E}}_{\text{t}}\text{S}{\text{O}}_{4}^{-}>\text{B}{\text{F}}_{4}^{-}\\ \approx \text{B}{\text{r}}^{-}>\text{OT}{\text{f}}^{-}>{\text{I}}^{-}>\text{SC}{\text{N}}^{-}≃\text{N}{\left(\text{CN}\right)}_{2}^{-}\gg \text{N}{\left(\text{Tf}\right)}_{2}^{-} \end{array}$$

The above ordering of ions remains valid at the dilute concentration of inorganic salts and ionic liquids; whereas, a higher concentration is required during actual processing that may lead an interchange of the ion position due to the secondary interactions at elevated concentrations. For example, [Chol]Cl has a denaturing effect both for lysozyme and α-lactalbumin only at a lower concentration as exhibited by the dip in their *T*_*m*_. The nature of [Chol]Cl toward lysozyme and α-lactalbumin start reversing at a higher concentration as suggested by their increasing *T*_*m*_ (Ajaj, 2010).

Klähn et al. studied the solvation stability of enzyme *Candida Antarctica* lipase B (CAL-B) in eight different ionic liquids and compared the results with that in water (Klähn et al., 2011a). The calculated solvation enthalpy indicated stronger interaction and hence lower solubility of the enzyme in ionic liquids than water. The lower solvation of CAL-B in ionic liquids is owing to the higher cavitation energy that further increases with IL-CAL-B interactions and subsequently denature the enzyme. The stronger interactions between the cation and anion lead to lower surface charge reduction from the surface of CAL-B as indicated by larger electrostatic potential values. Furthermore, charge density was more toward the polar surface of enzyme whereas the non-polar alkyl remains oriented toward the non-polar segment. During the investigation of CAL-B unfolding at a higher temperature in similar ionic liquids, Klähn et al. noted that hydrophobic ionic liquid causes higher stabilization of CAL-B than the hydrophilic ionic liquids (Klähn et al., 2011a). The interaction of CAL-B with ionic liquids was mainly at the polar surface and the non-polar core. The high polarizability of anion increases the extent of hydrogen bonding with the protein surface and destabilizes the CAL-B whereas the hydrophobic alkyl chain interacts with the CAL-B core. In the case of most hydrophobic cation acyclicbutylpentamethylguanidinium ([BAGUA]^+^), the protein core destabilizes due to the conformation change in the CAL-B. This exposes the protein core to the ionic liquids and stabilizes the unfolded protein. However, these observations could not be observed in the case of other hydrophobic ionic liquids due to the low solubility and weaker dispersion of protein. Fujita et al. reported the stabilizing role of biocompatible ionic liquids in solubilizing and stabilizing the cytochrome c (Fujita et al., 2005). As suggested by the study, the stabilizing role of biocompatible ionic liquids was bound to the dihydrogen phosphate (dhP). The addition of excess water has a negative destabilizing effect as revealed by the DSC spectra. An interesting, unfolding/refolding equilibria for lysozyme was noticed in a sugar solution of ethylammonium nitrate, EAN ([${\text{CH}}_{3}{\text{CH}}_{2}{\text{NH}}_{3}^{+}$][${\text{NO}}_{3}^{-}$]) with 97% refolding success by Byrne et al. (2007).

The experimental and theoretical studies indicated the dominance of anion in controlling the unfolding/folding equilibria (Haberler and Steinhauser, 2011; Haberler et al., 2011). The pronouncing role of the anion is attributed to the high polarizability that enables them to undergo coulombic and hydrogen bonding interactions at the protein surface also confirmed by the longer residence time (Haberler, M., and Steinhauser). Despite being the more influential in protein unfolding, anion approach to the positively charged surface of the protein in the form of ion-pair. The repulsive interaction between the surface and cation is compensated by the strong Coulomb interaction between the ions (Klähn et al., 2011b). The unfolding of the hen egg-white lysozyme (HEWL) in a dilute solution of EAN represents one such case where despite a strong coulomb interaction nearly 75% activity of HEWL was restored. The EAN was effective in high doses (1.6 mg/ml) compared to other renaturing agents (e.g., urea and guanidinium chloride) and the refolding efficiency increase up to 90% upon dilution (Summers and Flowers, 2000). The strong efficiency of EAN as a renaturating agent is because of its ability to extend hydrogen bonding network to the active sites similar to water.

#### Thermodynamics of the Hofmeister Series

**Figure 4:**
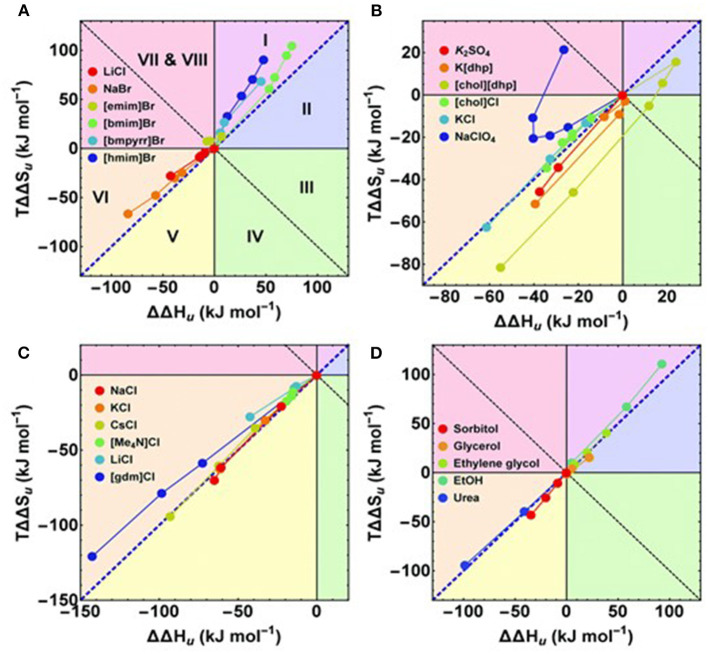
**(A–D)** Enthalpy–entropy compensation plots. The different segments correspond to different contributions of ΔΔ*G*_u_, ΔΔ*H*_u_, and TΔΔ*S*_u_. The blue diagonal corresponds to a complete enthalpy–entropy compensation. Data points correspond to different concentrations of the respective cosolute. **(A,C,D)** The first or the first two data points (≤ 0.5 M) of some compounds are omitted for clarity. Adopted from Senske et al. (2016).

Protein stability largely depends on the concentration of the ionic liquid. At lower ionic liquid concentration, say *c*_IL_ < 0.5 M, all ionic liquids behave as a denaturing agent for proteins. At *c*_IL_ > 1 M, ion-specific effects become dominant and follow Hofmeister ordering in protein stabilization (Senske et al., 2016). This shift in the behavior of ionic liquids is different than the inorganic salts mainly because of the diffused charges on ions. The folding/refolding behavior is reported as the change in melting temperature (Δ*T*_*m*_) upon transferring the protein from an ionic liquid solution to an ionic liquid-free buffer solution. At lower concentration, *c*_IL_ < 0.5 M, denaturation starts and results in negative (Δ*T*_*m*_). As the concentration increases beyond 1 M, renaturation starts in protein that brings a positive change in the Δ*T*_*m*_ values. However, a positive Δ*T*_*m*_ for [Chol][dhp] was observed even at 0.25 and 0.5 M. *T*_*m*_ is defined as the midpoint of the unfolding transition, where Δ*G*_u_ = 0. The cosolute-induced changes relative to the cosolute-free, buffered solution can be expressed as the excess functions (Equation 6),

(6) $$\begin{array}{l} \Delta \Delta G_{\text{u}}=\;\Delta G_{\text{u},\text{ IL}}-\;\Delta G_{\text{u},\text{ buffer}}=\Delta \Delta H_{\text{u}}-\;T\Delta \Delta S_{\text{u}} \end{array}$$

where, ΔΔ*G*_u_ is the excess Gibbs free energy and ΔΔ*H*_u_ and *T*ΔΔ*S*_u_ are the enthalpic and entropic contributions, respectively, that provide intriguing thermodynamic fingerprinting of the molecular mechanism involving a solute. Both ΔΔ*H*_u_ and *T*ΔΔ*S*_u_ are cosolute-dependent and favor either the direct or reverse Hofmeister ordering for ions. For the favorable unfolding at *c*_IL_ > 1 M, the *G*_u_ > 0. This trend in ΔΔ*G*_u_ is parallel to that of Δ*T*_*m*_ during unfolding of RNase A. An *enthalpy-entropy* compensation between ΔΔ*H*_u_ and *T*ΔΔ*S*_u_ is observed in case of analyzing the role of cosolute on protein unfolding. Based on the sign of excess function ΔΔ*G*_u_, ΔΔ*H*_u_ and *T*ΔΔ*S*_u_ eight possibilities are there as shown in Figure 4. According to Figure 4, hydrophobic ionic liquids (alkyl chain length from 4 to 6 and more carbons) have more positive *T*ΔΔ*S*_u_ than ΔΔ*H*_u_ leading to enthalpic stabilization and entropic destabilization. For hydrophilic salts, both *T*ΔΔ*S*_u_ and ΔΔ*H*_u_ were negative and the classification of ionic liquids into the stabilizing and destabilizing salts was depending on the magnitude of the relative values of these values. As the magnitude of thermodynamic parameters of cosolute-protein interactions depends on the pH, isoelectric point, charge on the protein and hydrophobicity, the arrangements of ionic liquid ions into the direct or reverse Hofmeister ordering is inconclusive. The increasing hydrophobicity of the cosolute promotes both the stabilizing enthalpic contribution and the destabilizing entropic contribution.

## Ionic Liquid-Deoxyribonucleic Acid (DNA) Interaction

Deoxyribonucleic acid (DNA) is a negatively charged, naturally occurring double-helical structure made up of phosphate groups, sugars, and nitrogenous bases. The DNA forms duplex *via* base-pairing in the cells and carries genetic information (Saenger, 1984). In the double-helical structure, nitrogenous bases project into the helix and are responsible for stabilizing the structure through a hydrogen bond, stacking- and charge-charge-interactions. The nucleotide base pairs in DNA are adenine-thymine (A-T) and cytosine-guanine (C-G). The A-T base pair stabilizes the double-helical structure of DNA less efficiently than the G-C base pair owing to the two hydrogen bonds in former than three in later (Yakovchuk et al., 2006). The two strands of DNA connects through phosphodiester bonds (Sinden, 1994). Owing to the phosphodiester bonds, sugars remain oriented in the same plane in the nucleotides and add more stability to the double-helical structure because of more van der Waals interactions. The nitrogenous bases exert a hydrophobic effect on the DNA structure and remain toward the cavity of helix while the phosphate and sugar groups are hydrophilic and orient toward the exterior side and interacts with water. The negatively charged phosphate groups are stabilized by the positively charged moieties for maximum stability. The phosphodiester bonds further add up to the strength of the electrostatic interactions by bringing the bases in close vicinity (Hunter, 1993). Apart from the number of A-T base pairs in the DNA, a base-pair mismatch during replication also affects the stability of DNA double helix. It has been observed that a T-C mismatch destabilizes the duplex by up to 5.8 kcal/mol. If a hydrogen bond stabilizes the double helix by 1.5 kcal/mol, the destabilization due to base-pair mismatch is way more than the stabilization and might cause structural distortion to the helix (Kool et al., 2000).

Depending on the choice of the solvent, nucleic acids [DNA and ribonucleic acid (RNA)] exist in three forms viz. A-, B-, and Z-form. RNA exists exclusively in the A-form while DNA in the B-form. However, under dehydrating conditions DNA exist in the A-form (Tateishi-Karimata and Sugimoto, 2014). The Z-form of DNA is highly unfavorable and noted only upon unfavorable base sequencing, under the high salt concentration, or the influence of a cation (Pan et al., 2014). Although the B-form of DNA is stable in water but owing to its large dependency on the electrostatic interactions; it undergoes denaturation upon changing the pH, temperature and ionic strength (Lerman, 1964; McFail-Isom and Sines, 1999; Bonner and Klibanov, 2000). Besides, co-solutes and osmolytes can also affect the stability of the DNA structure. The concentration of sodium and chloride ions attached to the DNA directly affects its stability as shown by the molecular dynamic simulation (Feig and Pettitt, 1999). The interaction of co-solutes with the unfolded DNA strands is used as a quantitative probe to reveal important information about the changes in structural conformation (Sasaki et al., 2007). In drug delivery application, drug molecules are used to perturb the DNA structure (Neidle et al., 1987). The stability of DNA is reflected in its melting temperature, *T*_*m*_. In the coming sections, we will discuss the effect of various ionic liquid systems on the stability, and extraction and purification of DNA.

### DNA Melting

The stability of DNA helix reflects in its melting temperature, *T*_*m*_. Because of the dependency of *T*_*m*_ on the polymerase chain reaction (PCR), any change in the double-helical structure during DNA hybridization can be accounted in terms of the *T*_*m*_ (Wittwer, 2009). The stability of the DNA double-helical structure upon rising the temperature changes depending on the mismatch in the DNA strands during hybridization and the strength of the buffer solution (Rouzina and Bloomfield, 2001; Gudnason et al., 2007). The *T*_*m*_ method can be used to account for the effect of solution conditions such as buffers, pH, hydrophobicity, solutes, analytes, or even surfactants. High salt concentration and high molecular weight polyethylene glycol were observed to stabilize the double-helical structure of DNA and, consequently, result in higher *T*_*m*_ (Khimji et al., 2013a). Cationic surfactants have been noticed to stabilize the DNA strands and therefore reduces the *T*_*m*_ (Bhattacharya and Mandal, 1997).

The excluded volume of a solvent is another factor that affects the DNA *T*_*m*_. The excluded volume is the free space of a solvent that can be occupied by another molecule. The salts containing polyanion possess large excluded volume than alkali halide salts viz. NaCl, NaBr, etc. Upon denaturation, DNA strands require a larger volume to expand than the duplex. In the presence of polyanions, which possess large excluded volume, the expansion of DNA duplex into the single stranded DNA gets thermodynamically less favored due to overcrowding and consequently, an increase in *T*_*m*_ is noticed (Khimji et al., 2013a).

### DNA Stability in Ionic Liquid Systems

The effect of ionic liquids on the structure of DNA and its subsequent stability has been a subject of major interest among researchers. Circular dichroism (CD) is normally used to assess any change in the DNA structure. An ionic liquid stabilizes DNA mainly through electrostatic bonding between the negatively charged phosphate group and the cation of the ionic liquid. At a higher concentration of ionic liquid, cation binds to several sites including phosphate and results in the destabilization of the structure. However, an adequate blend of specific and electrostatic interactions in ionic liquids has been noted to stabilize the structure more efficiently.

#### DNA Stability in Neat Ionic Liquids

The CD spectrum of DNA duplexes from salmon testes in choline dihydrogenphosphate ([ChCl][dhp]) exhibited only B-form of DNA. Besides, both quadruplexes (G-quadruplex and i-motifs) of DNA where noted stable in [ChCl][dhp], similar to water (Fujita and Ohno, 2010; Vijayaraghavan et al., 2010; Tateishi-Karimata et al., 2015). Thermodynamic parameters determination indicated abnormally higher stability of A-T base pairs than G-C base pairs (Watson-Crick base pairs) in 4 M [ChCl][dhp] solution compared to that in buffered NaCl solution due to the differences in the enthalpy contributions (Tateishi-Karimata and Sugimoto, 2012). Molecular dynamics (MD) and NMR results revealed choline binding to the minor groove of A-T base pairs in B-form of DNA and stabilize it via hydrogen bonding (Nakano et al., 2014a; Marusic et al., 2015). In the case of G-C base pairs, choline undergoes for specific binding with the guanine in the single strand of DNA that further inhibits the duplex formation via base pairing (Tateishi-Karimata and Sugimoto, 2012). This unusual behavior of ionic liquid solution toward A-T base pairs was more investigated further by experimental *in vitro* and MD *in silico* studies. In this succession, Chandran et al. observed that apart from the electrostatic interactions, groove binding of ionic liquid cation through hydrophobic and polar interactions significantly add up to the stability of DNA duplex (Chandran et al., 2012). The intrusion of DNA's minor groove by ionic liquid cation was further confirmed by the fluorescent intercalation dye replacement experiment. Portella et al. showed using MD simulations that G-C base pairs possess preferably higher solvation energy than the A-T base pairs in an ionic liquid solution and solvation differences greatly affect the DNA stability (Portella et al., 2014). It is concluded that the specific behavior of ionic liquid solution toward the DNA arises from the groove binding. The interaction of DNA with ionic liquid components are shown in Figure 1 and summarized in Tables 1, 2.

Zhang et al. showed that poly[3-butyl-1-vinylimidazolium L-proline salt] could condense plasmid DNA to form stable complexes against enzymatic degradation by deoxyribonuclease l (Zhang et al., 2009). Chen et al. studied the solubility and chain conformation of DNA in 1-allyl-3-methylimidazolium chloride ([Amim]Cl) and 1-butyl-3-methylimidazolium formate ([bmim][HCOO]) using laser light scattering (Chen et al., 2011). The DNA chain was observed in random conformation in [Amim]Cl and denatured and condensed in [bmim][HCOO]. Cardoso and Micaelo investigated the molecular solvation of single-stranded DNA (ssDNA) and double-stranded DNA (dsDNA) in detail in a variety of ionic liquids comprised of imidazolium, oxazolium, pyrrolidinium, pyrimidinium, quaternary ammonium, and choline as cation and tetrafluoroborate ([BF_4_]) and hexafluorophosphate ([PF_6_]) as counterpart anion (Cardoso and Micaelo, 2011). The modeling and MD simulation studies exhibited that dsDNA retains its B-form in all ionic liquids as shown by crystallographic data. The most stabilizing ionic liquids toward the dsDNA were based on choline and pyridine as cation and [PF_6_] as a binding anion. The [BF_4_] anion was observed to undergo stronger hydrogen bond formation than the [PF_6_]. The ssDNA was more accessible toward the hydrogen bonding *via* fluorinated anions. The cation of ionic liquid was located close to the DNA main chain owing to the electrostatic interactions with a phosphate group and hydrogen bonding and edge-to-face NH—π interaction with the bases, while anion forms hydrogen bonding with the cytosine, adenine and guanine bases. Mukesh et al. reported high solubility (3.5%) and long-term stability (up to 6 months) of B-form of DNA, obtained from salmon testes, in bio-based ionic liquid choline indole-3-acetate (Mukesh et al., 2013). Singh et al. reported long term storage capability (1 year) and high solubility (25 w/w%) of salmon tests DNA in 2-hydroxyethylammonium formate (2-HEAF) at ambient temperature (Singh et al., 2017). ITC results indicated the hydrogen bonding between DNA and 2-HEAF responsible for the high concentration solubility and extended stability. The DNA docking analysis exhibited a higher preference for minor-groove binding over DNA surface with 2-HEAF than the major-groove binding and thus confirming the importance of hydrogen bonding in stabilizing DNA. Sarkar and group investigated the role of hydrogen bonding in the DNA stabilization by employing ionic liquids, namely, 1,1,3,3-tetramethylguanidinium acetate (TMG) and 2,2-diethyl-1,1,3,3-tetramethylguanidinium acetate (DETMG), in which former has a hydrogen bonding N-H moiety whereas the latter has a different mode of bonding (Sarkar et al., 2020). MD simulation and spectroscopic results indicated that only the groove binding of ionic liquid to the DNA is not sufficient for stabilization of the structure. The TMG cation stabilized the Watson-Crick pair more efficiently than larger DETMG cation owing to the hydrogen bonding interaction differences with DNA grooves.

#### DNA Stability in Aqueous Ionic Liquids

As hydration itself has a major impact on DNA stability, ionic liquid as co-solvent in water has been explored as suitable media in DNA stabilization and storage. Wang et al. observed that [bmim][PF_6_] effectively extracts the salmon tests DNA from aqueous solution; in which [bmim]^+^ cation displace the Na^+^ from the phosphate group (Wang et al., 2007). In a further study, they noted that [bmim][PF_6_] replaces the ethidium bromide from the dsDNA as fortified by the lowering in the signals of the resonance light scattering (RLS) (Cheng et al., 2007). Khimji et al. contrarily observed the inefficiency of the hydrophobic ionic liquids [bmim][PF_6_] and 1-hexyl-3-methylimidazolium hexafluorophosphate ([hmim][PF_6_]) in extracting DNA from aqueous solutions despite their efficacy in extracting DNA staining dyes (Khimji et al., 2013b). Guo et al. examined the molecular mechanism and binding characteristics of DNA and [bmim]Cl in the aqueous medium (Ding et al., 2010). They reported a lowering in the critical aggregation concentration of [bmim]Cl in presence of DNA and a reduction in the fluorescence quenching of ethidium bromide upon addition of [bmim]Cl in DNA suggesting a competitive interaction between the dye, [bmim]Cl and DNA. Based on the experimental and quantum chemical results they proposed a DNA-ionic liquid interaction mechanism. At a concentration of [bmim]Cl below 0.06 M, the cationic head group locates at several Å of DNA phosphate with the alkyl chain lying parallel to the DNA surface, whereas, at higher concentrations (>0.06 M), the cationic head group situates nearer to the phosphate while butyl chain attached perpendicularly to the DNA surface. He et al. extensively studied the interaction between the 1-dodecyl-3-methylimidazolium bromide ([ddmim]Br) and DNA in dilute brine using isothermal titration calorimetry (ITC), micropolarity, dynamic light scattering, UV–Vis transmittance, atomic force microscopy (AFM), circular dichroism (CD) and molecular dynamics simulation (He et al., 2013). They observed strong electrostatic interaction between DNA and ionic liquid cation and hydrophobic interaction between the [ddmim]Br alkyl chains. Very high thermal stability (up to 100°C) and long-term storage stability (up to 6 months) observed for salmon tests DNA in [choline][lactate], [choline][H_2_PO_4_] (containing 20–50 wt% water) and [choline] [nitrate] (containing 20% water), as defined by CD and fluorescence spectra as well as gel electrophoresis.

Tateishi-Karimata et al. noted that DNA triplexes that are difficult to achieve in aqueous solution at neutral pH, can be stabilized in [ChCl][dhp] solution at neutral pH (Tateishi-Karimata et al., 2014). Surprisingly, the Hoogsteen base pairs and Watson-Crick base pairs exhibited similar stability in hydrated ionic liquid. MD simulation results of DNA triplex in [ChCl][dhp] by Nakano et al. indicated the groove binding of choline to the third strand of DNA (Nakano et al., 2014b). Stable G-quadruplexes were shown to occur in [ChCl][dhp] with choline location at the center of the quadruplex (Fujita and Ohno, 2012). Thermodynamic calculations coupled with MD simulation results showed higher stability of i-motifs than G-quadruplex in [ChCl][dhp] owing to the binding of the choline to the loops and grooves of i-motifs (Tateishi-Karimata et al., 2015). A detailed account of various studies involving ionic liquids and their influences on the DNA structure stability is given by Zhao (2015) and Tateishi-Karimata and Sugimoto (2018) groups.

### Extraction and Purification of DNA

The extraction, purification and storage of DNA are required for the biological experiments. The quantification of transcript and protein level of target genes Hyde and Read, 1993 is essential for understanding gene regulatory mechanisms and drug design that controls gene expression. The current liquid-liquid extraction methodology for the separation, purification and storage of nucleic acids involves highly toxic reagents such as phenol and chloroform; therefore, a simpler method involving an environmentally-benign solvent is highly sought (Tan and Yiap, 2009).

DNA is not stable in an aqueous medium at room temperature for a long period owing to the degradation by contaminating nucleases and inherent stability (Sasaki et al., 2007; Armand et al., 2009). Additionally, the lower vapor pressure of the water makes them unsuitable in the small scale operational methods as it quickly vaporizes; therefore, solvents that alleviate the problem of aqueous buffers are desirable for the development of functional devices (Armand et al., 2009). Because of the vaporless characteristic even at zero pressure, the extensive network of hydrogen bonding akin to water and coulombic nature, ionic liquids emerged as the most suitable alternative compared to the toxic organic solvents (Earle and Seddon, 2000). The solvation capability for both polar and apolar moieties and ease of tailoring makes ionic liquid a smarter solvent from the DNA-ionic liquid interaction perspective.

Shi et al. proposed a bicyclic imidazolium-based ionic liquid [b-4C-im][Br] for promoting the PCR signal of G-C rich DNA by minimizing non-specific amplifications (Shi et al., 2012). The ionic liquid also facilitated the PCR of normal-GC DNA under mild conditions because of the destabilization of DNA duplexes under mild conditions. Anderson group reported the efficiency of hydrophobic magnetic ionic liquids (MILs) in the extraction of long and short plasmid DNA from bacterial cell lysate and salmon tests (Clark et al., 2015a). Benzyltrioctylammoniumbromotrichloroferrate(III) ([(C_8_)_3_B_n_N^+^]-[FeCl_3_Br^−^]) MIL was efficient in selective extraction of smaller single-stranded and double-stranded DNA whereas the dicationic 1,12-di(3-hexadecylbenzimidazolium)dodecane bis[(trifluoromethyl)-sulfonyl]imide bromotrichloroferrate(III) ([(C_16_B_n_IM)_2_${\text{C}}_{12}^{2+}$][${\text{NTf}}_{2}^{-}$, FeCl_3_Br^−^]) MIL were excellent in extraction for the higher DNA molecules. In another report, MIL with metal-containing cations (Ni^2+^, Mn^2+^, or Co^2+^) and chloride anion were applied for *in situ* dispersive liquid–liquid microextractions (DLLME) for the extraction of long and short double-stranded DNA (Bowers et al., 2019). These hydrophilic MILs were further converted to hydrophobic MILs during the extraction by exchanging the chloride anion with that of bis[(trifluoromethyl)sulfonyl]imide anion to ease the separation. To make the whole process faster and sensitive, extraction methodology was coupled with anion-exchange high-performance liquid chromatography with diode array detection (HPLC-DAD) and fluorescence spectroscopy. The developed *in situ* MIL-DLLME method noted effective than the conventional DLLME method as it required only 3 min for DNA extraction and yielded 1.1–1.5 times higher extraction efficiency (EFs). In another attempt, the Anderson group developed a method to remove the DNA further from MIL by designing the PCR buffer and coupling it with the process (Clark et al., 2015b). Clark et al. further developed a particle-free approach to sequence-specific DNA extraction using a magnetic liquid support and ion-tagged oligonucleotide (ITO) probes that can distinguish nucleotide mismatch and was more sensitive than a commercial magnetic bead-based method for the capture of target DNA from a pool of interfering genomic DNA (Clark et al., 2017).

Another aspect of DNA extraction comes from the bacterial gene transformation that implies the gene cloning technology (Lorenz and Wackernagel, 1994). In this technology, bacteria receives a new genetic trait through foreign DNA. Such gene transformation occurs spontaneously in few prokaryotes naturally; however, most of the bacterial cells require artificial transformation, stemming from the slow diffusion of hydrophilic DNA's entry across the hydrophobic lipid bilayer membrane and the slight electrostatic repulsion due to anionic DNA as well as the anionic head-groups of the bilayer membrane (van Die et al., 1983). Also, DNA molecules undergo hydrolysis and enzymatic degradation during transformation (Caruso et al., 2012). During transformation, DNA molecules are loaded onto the delivery vectors that efficiently transfer the genetic material across the lipid bilayer without any possibility of enzymatic degradation. The DNA delivery vectors mainly involve the encapsulation of DNA within the delivery vectors such as cationic surfactants, triblock copolymer vesicles, viral capsids, protein superstructures, lipid assemblies, polymer nanocapsules, and mesoporous structure and are crafted within the self-assembled superstructures, followed by transformation studies (Kikuchi et al., 1999; Vijayanathan et al., 2002; Guo, 2005; Li et al., 2011; Mingozzi and High, 2011). In addition to this DNA delivery vehicle-mediated transformation, some physical techniques such as a biolistic method, microinjection, electroporation, and heat shock, and poly(ethylene glycol) also required during the transformation to increase the efficiency (Klebe et al., 1983; Smith et al., 1992; Amoozgar and Yeo, 2012). The well-established calcium chloride method proposes that Ca^2+^ binds with anionic DNA and gets transferred via the transient pores formed during the heat shock. The necessity of heat shock and the inability to induce self-assembly in DNA during transformation renders calcium chloride method less lucrative. Instead, involving a hydrophobic ionic liquid that contains organic cation and also provides electrostatic interaction owing to the oppositely-charged ions and induces DNA self-assembly during the transformation. Soni et al. reported the formation of novel functional nanostructures during the electrostatic interaction between the phosphate group of DNA and the cationic part of hydrophobic ionic liquid [bmim][PF_6_] (Soni et al., 2015). The self-assembling nanostructures acted as promising synthetic non-viral vectors for the efficient bacterial pGFP gene transformation in cells. TGA analysis of the DNA-IL functional nanostructures revealed that nanostructures consist of about 16 wt% ionic liquid, which may stabilize the pDNA and eventually enhance transformation efficiency. Samarkina et al. studied the behavior of supramolecular systems based on homologous series of amphiphiles bearing imidazolium fragment (C_n_H_2n_+1Im^+^Br^−^, where *n* = 14, 16, 18) toward the aggregation behavior, solubilization activity toward the hydrophobic guest, interaction with DNA decamer as well as integration with the lipid bilayer (Samarkina et al., 2017). The elongation of hydrophobic moiety by two methylene groups was noted to decrease critical micelle concentration by 4-folds without any change in the solubility. The ability of amphiphiles to integrate with lipid bilayer strongly depends on the length of the hydrophobic fragments. The lower homolog C_14_H_29_Im^+^Br^−^ increases the permeability of lipid bilayer whereas the higher homologs C_16_H_33_Im^+^Br^−^ and C_18_H_37_Im^+^Br^−^ stabilize it. Serker et al. noted positive zeta potential when employed [bmim][PF_6_] for plasmid DNA transformation (Sarker et al., 2019). [bmim][PF_6_] protected the plasmid DNA against ultrasonic shear stress and also enhanced *in vitro* gene transfection efficiency.

## Toxicity and Recyclability of Ionic Liquids

The enormous interests in ionic liquids as biostabilizers necessitates the documentation of their toxicity on the environmental systems and various organisms. The toxicity of different ionic liquids is summarized by Thuy Pham et al. (2010). The inhibitory action of various ionic liquids on the activity of enzyme acetylcholinesterase is reported by many workers (Stock et al., 2004; Jastorff et al., 2005; Matzke et al., 2007; Ranke et al., 2007; Arning et al., 2008). The enzyme acetylcholinesterase has a major role in nerve response and function. The repressive action of ionic liquids on the activity of acetylcholinesterase has been observed to arise from the cation (Arning et al., 2008). However, later, several studies suggested that the toxicity depends on both cation and anion (Thuy Pham et al., 2010). The measurements showed that pyridine-based ionic liquids were less toxic than the imidazolium- and phosphonium-based ionic liquids. Contrary to the cations, anions are non-inhibitory to the enzyme activity except those containing fluoride and complex anions of fluorine ([BF_4_]^−^, [PF_6_]^−^, [OTf]^−^, [NTf_2_]^−^, [SbF_6_]^−^) (Stolte et al., 2006). The hydrolysis of complex anions of fluorine produces hydrofluoric acid which is a potential inhibitor of Na^+^-K^+^-ATPase, at the cell surface, and may interfere with essential cellular processes. The non-inhibitory action of anions might arise because of the binding to active site of enzyme (Matzke et al., 2007). However, the presence of alkyl chain on cation or anion increases the toxic effect of ionic liquids. The hydrophobicity of ionic liquids is also indicative of its toxicity (Kumar et al., 2009). The hydrophobicity of ionic liquids mainly arises due to the low charge density of anions. It is linked with the presence of fluorine atom in anion and increase with the number of fluorine atoms. However, a comparison between the alkyl chain length and anion in affecting the toxicity showed that the alkyl chain lengths on the cation gave rise to a larger impact on toxicity than a fluorinated anion. The effect of fluorinated anion ([NTf_2_]^−^) vanishes as the alkyl chain length reaches beyond octyl. Similarly, the unsaturation in cationic core and alkyl group on cation promotes toxicity (Kumar et al., 2009). Despite the distinct effect of different features of ionic liquids on toxicity, ionic liquids are noted as less toxic than their precursors (Kumar et al., 2009). Pyrrolidinium- and piperidinium-based ionic liquids containing propyl and butyl alkyl chain and bromide as the counter anion are less toxic than pyrrolidine and piperidine, respectively (Kumar et al., 2009). The task-effective ionic liquids containing nitrile, dimethyldisulphide, hydroxy, and polar ether group on cation reduces the toxicity compared to the non-functionalized ionic liquids (Stolte et al., 2006; Kumar et al., 2009). Thus, aprotic ionic liquids without modification cannot be considered as biocompatible ionic liquids due to their toxicity.

The protic ionic liquids have been shown to possess low cytotoxicity due to the lack of hydrophobic cation and perfluorinated anion (Gouveia et al., 2014). Choline-based protic ionic liquids are excellent example of biocompatible ionic liquids due to the low toxicity of choline and hence, have been involved as stabilizers for biopharmaceuticals (Gouveia et al., 2014). Weaver et al. studied the toxicity of choline-based protic ionic liquids combined with phosphate-based anions. The [Chol][DHP] exhibited the lowest cytotoxicity. Thus, choline family is one of the most suitable candidates of biocompatible protic ionic liquids and can be utilized as medium for protein and DNA stabilization. The development of biocompatible ionic liquids makes the toxicity issue irrelevant for their use as medium in many therapeutic applications and as stabilizers for biomolecules.

The wide applicability of ionic liquid systems in biomolecules stabilization, purification and storage makes them the frontrunner in the quest of the sustainable solvents. However, the efficient recyclability and reuse of ionic liquids are required to reach the economic and environmental goals. The recovery of ionic liquids is also essential as these solvents are toxic and non-biodegradable and releasing them to the aquatic environment might cause sever contamination (Frade and Afonso, 2010). These are the prime issues while using ionic liquids at a large scale. Several methods have been proposed to recover ionic liquids from solution, namely, distillation, adsorption, membrane separation, ion-exchange, and liquid-liquid extraction processes. The choice of the method in recovery depends on the medium characteristics, nature, and concentration of ionic liquids.

The negligible vapor pressure of ionic liquids makes their recovery easy from the ionic liquid-solvent mixtures by evaporation. However, a large difference in their boiling point is needed for adopting this method. Hydrophobic ionic liquids are easier to separate from aqueous mixture than hydrophilic one because of their water-immiscible nature. Membrane separation is a well-known, cost-effective, and commercially applicable method of separation for biomolecules (Haerens et al., 2010). In membrane separations, a membrane capable of separation retains specific compounds while allowing the passage to others. In pressure-driven membrane separation processes *viz*. microfiltration, ultrafiltration, nanofiltration, etc., when a pressure is applied across the membrane, the feed stream is split into permeate and retentate. Ionic liquids can be separated from permeate. Ultrafiltration method is used widely to separate ionic liquids from the proteins. Ultrafiltration membranes have a pore size 2–100 nm that allows the ionic liquid solution to pass through it while retaining the protein (Van Der Bruggen et al., 2003). The recovery of ionic liquids by ultrafiltration can be further improved by tuning the flow rate, concentration ratio, and temperature as suggested by Liu and Wu (1998).

Ionic liquid recovery from the DNA does not require special techniques. Precipitation of both DNA and ionic liquid upon addition of suitable solvents followed by the filtration and evaporation is normally used. In general, an ice-cold isopropyl alcohol (IPA)/ethanol is added to the ionic liquid-DNA mixture to precipitate the DNA (Mukesh et al., 2013; Singh et al., 2017). Upon adding ethyl acetate, the ionic liquid separates at the bottom and remaining DNA forms a hazy layer at the top that becomes transparent in 3–4 h. The traces of DNA can be completely removed from the ionic liquid by applying the same treatment again.

## Conclusions and Outlook

Despite the large volume of research on ionic liquids in various physical and chemical transformations, their employment in the area of biochemistry has only seen growth in the last two decades. The remarkable properties such as insignificantly low vapor pressure and structure-tunability render ionic liquids more superior over the hazardous volatile organic solvents (VOSs) and, therefore, can be employed as a potent medium in controlling the functionality and stability of biomolecules like protein and DNA. Protein solubility, structural stability, crystallization, and separation in ionic liquids are often scaled either in the direct or indirect order of the Hofmeister ordering of ions. However, the overall influence of a particular ionic liquid on a protein cannot be predicted and necessitates further investigation both from the experimental and computational researches. Compared to the dubious conclusions drawn from the ionic liquid-protein interactions owing to the structural variations, ionic liquid-DNA interactions are facile as the expected outcome of a particular ionic liquid on the structure and stability can be foreseen based on the available research. In the case of DNA, ionic liquids emerged as a natural long-term stabilizer and nuclease inhibitor that causes slow degradation to the stored DNA. Ionic liquids provide the five major interacting forces like hydrogen bonding, base stacking, conformational entropy, hydration, and cation binding that determine the structure and stability of ionic liquids. Despite the growing interests of ionic liquids in biochemistry, their moderate toxicity and involvement of volatile organic compounds during synthesis and recyclability are foremost concerns lingered with these coulombic media and ought to be addressed shortly. However, the introduction of biocompatible ionic liquids eliminates the problem of toxicity up to a certain length.

## Author Contributions

All authors have made significant contributions to the reading, writing sections of the manuscripts, and approved the final version submitted to the journal.

## Conflict of Interest

The authors declare that the research was conducted in the absence of any commercial or financial relationships that could be construed as a potential conflict of interest.
